# Comprehensive Characterization and Validation of Chromosome-Specific Highly Polymorphic SSR Markers From Pomegranate (*Punica granatum* L.) *cv.* Tunisia Genome

**DOI:** 10.3389/fpls.2021.645055

**Published:** 2021-03-16

**Authors:** Prakash Goudappa Patil, Nripendra Vikram Singh, Abhishek Bohra, Keelara Puttaswamy Raghavendra, Rushikesh Mane, Dhananjay M. Mundewadikar, Karuppannan Dhinesh Babu, Jyotsana Sharma

**Affiliations:** ^1^ICAR-National Research Centre on Pomegranate, Solapur, India; ^2^ICAR-Indian Institute of Pulses Research, Kanpur, India; ^3^ICAR-Central Institute for Cotton Research, Nagpur, India

**Keywords:** chromosome, genome, hypervariable SSR, pomegranate, polymorphism

## Abstract

The simple sequence repeat (SSR) survey of ‘Tunisia’ genome (296.85 Mb) identified a total of 365,279 perfect SSRs spanning eight chromosomes, with a mean marker density of 1,230.6 SSRs/Mb. We found a positive trend in chromosome length and the SSR abundance as marker density enhanced with a shorter chromosome length. The highest number of SSRs (60,708) was mined from chromosome 1 (55.56 Mb), whereas the highest marker density (1,294.62 SSRs/Mb) was recorded for the shortest chromosome 8 (27.99 Mb). Furthermore, we categorized all SSR motifs into three major classes based on their tract lengths. Across the eight chromosomes, the class III had maximum number of SSR motifs (301,684, 82.59%), followed by the class II (31,056, 8.50%) and the class I (5,003, 1.37%). Examination of the distribution of SSR motif types within a chromosome suggested the abundance of hexanucleotide repeats in each chromosome followed by dinucleotides, and these results are consistent with ‘Tunisia’ genome features as a whole. Concerning major repeat types, AT/AG was the most frequent (14.16%), followed by AAAAAT/AAAAAG (7.89%), A/C (7.54%), AAT/AAG (5.23%), AAAT/AAAG (4.37%), and AAAAT/AAAAG (1.2%) types. We designed and validated a total of 3,839 class I SSRs in the ‘Tunisia’ genome through electronic polymerase chain reaction (ePCR) and found 1,165 (30.34%) SSRs producing a single amplicon. Then, we selected 906 highly variable SSRs (> 40 nt) from the ePCR-verified class I SSRs and *in silico* validated across multiple draft genomes of pomegranate, which provided us a subset of 265 highly polymorphic SSRs. Of these, 235 primers were validated on six pomegranate genotypes through wet-lab experiment. We found 221 (94%) polymorphic SSRs on six genotypes, and 187 of these SSRs had ≥ 0.5 PIC values. The utility of the developed SSRs was demonstrated by analyzing genetic diversity of 30 pomegranate genotypes using 16 HvSSRs spanning eight pomegranate chromosomes. In summary, we developed a comprehensive set of highly polymorphic genome-wide SSRs. These chromosome-specific SSRs will serve as a powerful genomic tool to leverage future genetic studies, germplasm management, and genomics-assisted breeding in pomegranate.

## Introduction

Pomegranate (*Punica granatum* L.) is an economically important perennial fruit crop (Patil et al., 2020c). The popularity of pomegranate worldwide stems from its nutritional values, specific organoleptic characteristics, and a variety of health benefits (Filannino et al., 2013; Teixeira da Silva et al., 2013). This crop has originated from Iran and is widely cultivated in drier parts of Southeast Asia, Iran, China, Japan, the United States (California), West Indies, tropical America, and India (Holland and Bar-Ya’akov, 2014). It is considered an excellent fruit crop for arid zones owing to its drought tolerance. Now, it is widely cultivated in Mediterranean, tropical, and subtropical regions (Holland and Bar-Ya’akov, 2008; Chandra et al., 2010). Its adaptation to the Mediterranean climate has led to its wide diffusion and the creation of a multitude of new genetic individuals over time (Tinebra et al., 2021). Therefore, Mediterranean and Middle-East countries are currently the main regions of pomegranate cultivation and production (Jbir et al., 2008; Melgarejo et al., 2009). Globally, India stands first in pomegranate cultivation with an area of 2.46 hundred thousand hectare, and production and productivity of 27.91 hundred thousand metric tonnes and 12 tonnes/ha, respectively (NHB, 2018).

Efforts to improve pomegranate through standard breeding practices have led to the development and release of few improved varieties in India (Jalikop et al., 2005). However, there is an urgent need to improve the efficiency of current breeding programs to deliver higher genetic gains. In this context, appropriate DNA marker technology facilitates the identification of genetic determinants (genes/QTLs) underlying various traits of economic significance (Patil et al., 2020b, c). Among the various marker techniques, microsatellites or simple sequence repeat (SSR) markers represent one of the most informative, abundant, and easy-to-use marker systems for genetic studies and plant breeding programs (Bohra et al., 2011, 2012; Ravishankar et al., 2013; Xu et al., 2013; Abd Ei-Moghny et al., 2017). Recently, three major classes of SSRs were reported in eggplant genome based on the SSR motifs length and repeats, which included class I (hypervariable: > 30 nt), class II (potentially variable: 20–30 nt), and class III (variable: < 20 nt) types (Portis et al., 2018). Earlier, Temnykh et al. (2001) also highlighted the significance of SSR tract lengths for marker development and breeding in rice. The rationale for such categorization is that the SSRs with greater tract length have shown higher degree of polymorphism in human (Weber, 1990; Xu et al., 2000). Similar research in various crops including rice, pigeonpea have shown significantly higher polymorphism of class I SSRs (> 20 bp) and highly variable SSRs (> 40 bp) (Singh et al., 2012; Dutta et al., 2013; Bohra et al., 2017).

Sequencing of multiple genomes enabled by the diverse array of sequencing technologies has paved the way for large-scale DNA markers. Discovery and characterization of the DNA markers spanning entire genome such as SSRs provides the foundation for trait discovery studies and molecular breeding. Following genome sequencing, genome-wide SSRs were designed for several plant species, including rice (Zhang et al., 2007), soybean (Song et al., 2010), *Brachypodium* (Sonah et al., 2011), maize (Xu et al., 2013), foxtail millet (Pandey et al., 2013), *Brassica* (Shi et al., 2014), cotton (Wang et al., 2015), *Nicotiana* (Wang et al., 2018), peanut (Zhao et al., 2017; Lu et al., 2019), eggplant (Portis et al., 2018), carrot (Uncu and Uncu, 2020), and more recently in pomegranate (Patil et al., 2020c).

In pomegranate, SSRs have been extensively employed to study genetic diversity and understanding population structure and association analysis (Curro et al., 2010; Pirseyedi et al., 2010; Singh et al., 2015). However, majority of these studies have reported low level of SSR polymorphism. Paucity of highly polymorphic and chromosome-specific molecular markers in pomegranate has hampered map-based gene/QTL strategy. In view of this, the present study aimed at developing a comprehensive set of chromosome-specific hypervariable SSR markers in pomegranate, which would be of immense utility for the pomegranate research and breeding. Availability of chromosome-level genome assembly of the pomegranate cultivar such as ‘Tunisia’ (Luo et al., 2019) provided us with unprecedented opportunity for genome-wide characterization and development of the first set of chromosome-specific highly polymorphic SSR markers.

## Materials and Methods

### Retrieving Genome Sequences

High-quality genome assembly with eight pseudo-chromosome molecules of pomegranate cultivar ‘Tunisia’ (Luo et al., 2019) was retrieved in FASTA format from the NCBI^1^. Three other draft genomes of pomegranate cultivars Dabenzi, Taishanhong, and AG2017 (Akparov et al., 2017; Qin et al., 2017; Yuan et al., 2018) were also retrieved for validation of identified SSRs using electronic polymerase chain reaction (ePCR).

### Chromosome-Specific Survey for SSR Motifs and Primer Design

Genomic sequences of eight ‘Tunisia’ pseudo-chromosome molecules, 296.85 Mb (excluding unknown 23.49 Mb sequences), were surveyed for the presence of chromosome-specific perfect, compound, and imperfect SSR repeats using Krait:ultra-fast SSR search module (Du et al., 2018). As criteria, only two to six nucleotide motifs were considered, and the minimum repeat unit was defined as 12 for mononucleotides, six for dinucleotide repeats, four for trinucleotide repeats, three for tetranucleotides and pentanucleotides, and two for hexanucleotides. Compound microsatellites were defined as two microsatellites interrupted by 100 bases.

The chromosome-specific hypervariable SSR motifs (> 30 bp) were first considered from the total list of motifs for each chromosome by using search options provided in Krait software, followed by selecting all the class I SSRs motifs (> 30 bp). Primer designing was performed using Primer module, which is implemented in Krait software. Primers were designed to generate amplicons of 100–400 bp in length with the following parameters: primer length (bp) 18–20, with 19 as the optimum; GC content 40–70%; Tm 52–60°C, with 55 as the optimum. The other parameters used were as that of default program values. All the genome-wide designed class I primers were designated as hypervariable SSR markers ‘Tunisia’ (HvSSRT).

### Creating Chromosome-Specific Marker Distribution Graphs and Physical Maps

Preliminary information was generated for all the SSR loci such as start and end positions on each chromosomes, and their major classes, i.e., classes I, II, and III using Krait software. Circular graph was drawn to depict chromosome-wise distribution of each SSRs by using the software ShinyCircos (Yu et al., 2017). Apart from this, based on physical positions and tract length of class I highly variable SSRs (> 40 nt), chromosome-specific scatter plots were drawn using Microsoft Excel. Finally, based on the information on physical positions of highly variable SSRs, the saturated SSR marker-based physical map of each chromosome was drawn using MapChart v 2.2 software (Voorrips, 2002).

### *In silico* Evaluation of Newly Developed SSRs for Polymorphism Through ePCR

To evaluate amplification efficiency of newly designed class I SSRs (> 30 nt) and to map the designed marker to genomic sequences of eight chromosomes of ‘Tunisia,’ the Genome-Wide Microsatellite Analyzing Tool Package (GMATA) software (Wang and Wang, 2016) was used to perform an *in silico* amplification by calling the ePCR algorithm (Schuler, 1997). The ePCR result was used to process the marker mapping information. The settings for ePCR were margin 3,000, no gap in primer sequence, no mismatch in primer sequence, allowed size range of 100–1,000, word size (-w) 12, and contiguous word (-f) 1. The output file (.emap) provided the detailed amplification patterns of the markers with calculated amplicon sizes and target positions on chromosomes and identified single-locus and multi-locus markers. Subsequently, highly variable SSRs (> 40 nt) were evaluated on eight chromosomes of ‘Tunisia’ to identify primers producing single amplification products. Finally, all the identified single-locus SSR primers of ‘Tunisia’ chromosomes were evaluated across the three draft genome sequences of pomegranate *cv.* Dabenzi, Taishanhong, and AG2017. The approximate amplicon sizes obtained for highly variable SSRs across the four pomegranate genomes using GMATA were used to calculate various marker parameters like number of alleles (Na), number of effective alleles (Ne), major allelic frequency (MAF), Shannon’s information index (*I*), observed heterozygosity (*Ho*), expected heterozygosity (*He*), and polymorphism information content (PIC) using GenAlEx v. 6.5 (Peakall and Smouse, 2012) software.

### Wet-Lab Validation of Developed SSR Markers

Genomic DNA was extracted from the fresh leaf samples of 30 pomegranate genotypes (Table 1) following the modified CTAB method (Ravishankar et al., 2000). For PCR experiment, a total of 235 chromosome-specific highly variable HvSSRT primer pairs were synthesized and initially screened on a subset of six pomegranate genotypes, i.e., ‘Bhagawa,’ ‘Daru 17,’ ‘Mridula,’ P-23, IC 318728, and IC 318790, following touch down and normal PCR conditions for different primers (Supplementary Table 5) with Prime-96^TM^ Thermal Cycler (Himedia, India). Amplicons were then scored on fragment analyzer QIAxcel Advanced (Qiagen India Pvt. Ltd.) and analyzed using QIAxcel Screen Gel Software. Subsequently, 16 HvSSRTs showing clear amplifications were selected randomly from eight chromosomes of ‘Tunisia’ for genetic diversity study in 30 pomegranate genotypes. For PCR experiments, amplification was carried out in 10 μl reaction volume containing 1.0 μl of 10 × PCR buffer, 1 μl (1 mM dNTP mix), 0.5 μl each of forward and reverse primers (10 pmol), 0.2 μl of *Taq* DNA polymerase 5U/ μl (Himedia, India), and 1 μl (10 ng) of template DNA. Touchdown PCR was performed with the following conditions: 94°C for 5 min, followed by 16 cycles of 94°C for 30 s, decrease 0.2°C/cycle from 60°C for 30 s, 72 C for 45 s, followed by 20 cycles of 94°C for 30 s, 55°C for 30 s, 72°C for 45 s, and a final extension at 72°C for 5 min. For normal PCR, initial denaturation at 94°C for 5 min, followed by 36 cycles of 94°C for 1 min, 55°C for 1 min, 72°C for 2 min, and a final extension at 72°C for 7 min was followed. PCR products were separated on 3% metaphor agarose gels, visualized, and photographed in gel documentation system (Vilbert Dourmet, France).

**TABLE 1:** Pomegranate genotypes used in the study.

**Sl. No.**	**Genotype name**	**Type**	**Origin/source**
1	Kalpitiya	Exotic cultivar	Sri Lanka
2	Bassein seedless	Cultivar	India (Karnataka)
3	Bhagawa	Commercial cultivar	India (Maharashtra)
4	Muscat	Exotic cultivar	Oman
5	GR Pink	Exotic cultivar	Russia
6	Ruby	Commercial cultivar	India (Karnataka)
7	P-26	Commercial cultivar	India (Maharashtra)
8	Yercaud HRS	Cultivar	India (Tamil Nadu
9	Ganesh	Commercial cultivar	India (Maharashtra)
10	Arakta	Commercial cultivar	India (Maharashtra)
11	Mridula	Commercial cultivar	India (Maharashtra)
12	P-16	Commercial cultivar	India (Maharashtra)
13	G-137	Commercial cultivar	India (Maharashtra)
14	Gulesha Red	Exotic cultivar	Russia
15	Jodhpur Red	Cultivar	India (Rajasthan)
16	KRS	Local collection	India (Karnataka)
17	P-23	Commercial cultivar	India (Maharashtra)
18	Jyoti	Commercial cultivar	India (Karnataka)
19	Kandhari	Exotic breeding line	Afghanistan
20	P-13	Commercial cultivar	India (Maharashtra)
21	Daru-17	Wild collection	India (Himachal Pradesh)
22	IC 1181	Wild collection	India (Uttaranchal)
23	IC 1194	Wild collection	India (Uttaranchal)
24	IC 318703	Wild collection	India (Himachal Pradesh)
25	IC 318720	Wild collection	India (Himachal Pradesh)
26	IC 318735	Wild collection	India (Himachal Pradesh)
27	IC 318753	Wild collection	India (Himachal Pradesh)
28	IC 318754	Wild collection	India (Himachal Pradesh)
29	IC 318779	Wild collection	India (Himachal Pradesh)
30	IC 318790	Wild collection	India (Himachal Pradesh)

### Genetic Diversity Analysis

The genotypic data of 30 test genotypes were used for estimating the genetic diversity parameters using GenAlEx v. 6.5 (Peakall and Smouse, 2012), the Na, Ne, *I*, *Ho*, *He*, and PIC. Frequency distribution graphs for allele number and PIC values were drawn using Microsoft Excel. The unweighted pair group method with an arithmetic mean (UPGMA)-based neighbor-joining tree (NJ) and principal coordinate analysis (PCoA) was performed using DARwin v. 6.0.13 (Perrier and Jacquemoud-Collet, 2006).

## Results

### Genome-Wide Discovery of SSRs

The SSRs were surveyed in the available genome assembly of pomegranate cultivar Tunisia for the presence of mono- to hexanucleotides having a tract length of ≥ 12 bp. As a result, a total of 365,279 perfect SSRs were identified from the 296.85 Mb of genomic sequences (Table 2). A total of 55,836 (15.28%) belonging to compound SSR category were detected. The hexanucleotide repeats were the most abundant (201,501) with a representation of 55.16%, followed by di- (55,437, 15.18%), tetra- (36,455, 9.98%), tri- (29,940, 8.2%), mono- (27,536, 7.54%), and penta (14,410, 3.94%) nucleotide repeats (Table 2).

**TABLE 2:** Characterization of microsatellites in the pomegranate genome *cv.* Tunisia.

**SSR mining**			**Total**	
Examined sequences size (bp)			296,847,911	
Total number of perfect SSRs			365,279	
Total length of perfect SSRs (bp)			5,177,752	
Relative abundance of SSRs (loci/Mb)			1,230.6	
Relative density for SSRs (bp/Mb)			17,443.55	
Total number of compound SSRs			55,836	
**Motif**	**SSR counts and percentage**	**Length (bp)**	**Relative abundance (loci/Mb)**	**Relative density (bp/Mb)**
Mono	27,536 (7.54%)	419,278	92.77	1,412.52
Di	55,437 (15.18%)	1,067,314	186.76	3,595.72
Tri	29,940 (8.2%)	466,194	100.87	1,570.58
Tetra	36,455 (9.98%)	485,912	122.81	1,637.01
Penta	14,410 (3.94%)	237,540	48.55	800.26
Hexa	201,501 (55.16%)	2,501,514	678.85	8,427.46
Total	365,279 (100%)	5,177,752		

The frequency distribution of different SSR motifs in the ‘Tunisia’ genome sequence is presented in Supplementary Figure 1. Among them, AT/AG had the highest occurrence (14.16%), followed by AAAAAT/AAAAAG (7.89%), A/C (7.54%), AAT/AAG (5.23%), AAAT/AAAG (4.37%), and AAAAT/AAAAG (1.2%) (Supplementary Figure 1). Across mono- to hexanucleotide repeats, the major motifs were A, AT, AAT, AAAT, AAAAT, and AAAAAT, of which AT motif had relative abundance of 120.6 loci/Mb in the genome, followed by A (80.3 loci/Mb) and AG (53.7 loci/Mb) motifs. SSRs with CG-rich repeats were rare in the pomegranate genome. We found an inverse relationship between SSR abundance and motif repeat number, and the trend was the most conspicuous for hexa- and tetranucleotide repeats (Supplementary Figure 4).

### The Intra-Chromosomal Distribution of SSRs

We further analyzed the distribution of SSRs on each chromosome (Table 3). We identified a total of 365,279 perfect SSRs in comparison with 415,716 imperfect SSRs with mean marker densities of 1,230.6 and 1,400.5 (SSR/Mb), respectively. The highest number of SSRs (60,708 perfect, 67,141 imperfect) was assigned to the largest chromosome 1 (55.56 Mb) followed by SSRs mapped onto chromosomes 2 (44.57 Mb, 56,038 perfect, 64,041 imperfect) and 4 (40.13 Mb, 51,511 perfect, 58,934 imperfect). Less perfect (35,868, 37,304, and 36,241) as well as imperfect SSRs (41,094, 42,809, and 41,901) were assigned to shorter chromosomes Chm_6 (28.33 Mb), Chm_7 (28.78 Mb), and Chm_8 (27.99 Mb), respectively. Therefore, chromosome length showed relation with the SSR abundance per chromosome in our study. The differences in SSR densities on different chromosomes were significant, ranging from 1,092.77 SSRs/Mb (Chm_1) to 1,294.62 SSRs/Mb (Chm_8), with an average of 1,230.60 SSRs/Mb (Table 3). It was also interesting to note that the marker density increased with the reduced chromosomes length (Supplementary Figure 2A).

**TABLE 3:** The chromosome-wise distribution of perfect, compound, and imperfect SSRs.

**Chromosome**	**Total Mb**	**Perfect**								**Compound**		**Imperfect**	
		**Mono**	**Di-**	**Tri-**	**Tetra-**	**Penta-**	**Hexa-**	**Total**	**SSRs/Mb**	**Total**	**SSRs/Mb**	**Total**	**SSR/Mb**
*Chm_1	55.56	4,289	8,574	4,842	6,141	2,275	34,587	60,708	1,092.77	9,090	163.62	67,141	1,208.57
Chm_2	44.57	4,278	8,617	4,518	5,459	2,241	30,925	56,038	1,257.43	8,573	192.37	64,041	1,437.01
Chm_3	39.96	3,486	7,027	3,944	4,863	1,933	26,189	47,442	1,187.40	7,138	178.65	53,647	1,342.70
Chm_4	40.13	4,082	8,041	4,187	5,072	2,008	28,121	51,511	1,283.75	8,036	200.27	58,934	1,468.74
Chm_5	31.53	3,053	6,186	3,377	4,006	1,637	21,908	40,167	1,273.87	6,115	193.93	46,149	1,463.58
Chm_6	28.33	2,746	5,419	3,048	3,701	1,427	19,527	35,868	1,266.29	5,506	194.38	41,094	1,450.79
Chm_7	28.78	2,933	5,694	2,951	3,685	1,487	20,554	37,304	1,296.21	5,716	198.62	42,809	1,487.50
Chm_8	27.99	2,669	5,879	3,073	3,528	1,402	19,690	36,241	1,294.62	5,662	202.26	41,901	1,496.81
Total	296.85	27,536	55,437	29,940	36,455	14,410	201,501	365,279	1,230.60	55,836	188.11	415,716	1,400.52

*Chm-, chromosome.

The intra-chromosomal distribution of SSR motif types in the ‘Tunisia’ genome as a whole (Supplementary Figure 2B), reflected the abundance of hexanucleotide repeats and the least occurrence of penta-/mononucleotide repeats. The distribution of motifs within each chromosome followed the pattern observed in the genome assembly as a whole, and hexanucleotides were the most frequent SSR types followed by dinucleotides in every chromosome. The hexa- (55.05%) and dinucleotide (15.24%) exhibited the highest level of variation among chromosomes, within Chm_1 showing the lowest percentage for di- (14.12%) and the highest for hexanucleotides (56.97%), while in Chm_8, we found higher percentages for di- (16.22%) and hexanucleotides (54.33%). Considering frequency distribution on a chromosome-by-chromosome basis, AT was found to be the most abundant dinucleotide motif followed by ‘A’ mononucleotide repeat. Similarly, AAT was the most prominent among trinucleotide repeat category, while AAAG was the most abundant tetranucleotide repeat (as in the genome as a whole), except for the Chr_7 (AATT). However, penta- and hexanucleotides showed two different motif combinations for each chromosome (Supplementary Figure 5).

### Chromosome-Specific Distribution of Three Major Classes of SSRs

**FIGURE 1:**
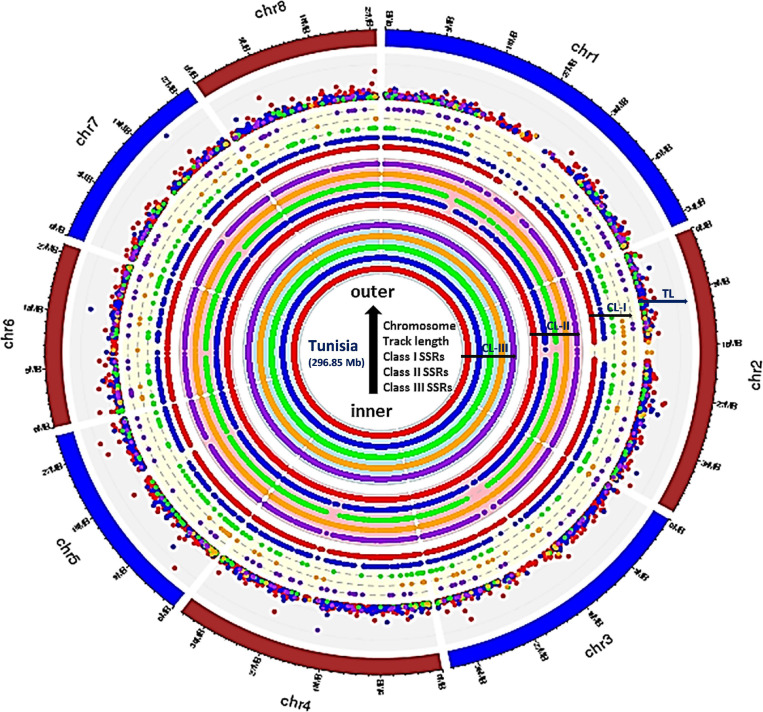
Circos graph depicting the chromosome-wide distribution for three major classes of perfect SSRs (excluding mononucleotides). Inner ring represents class III, whereas middle and outer rings correspond to class II and class I, respectively, and the densely placed colored dots depict the tract length for each motif types. All rings from inside to outside have five subrings representing, di-, tri-, tetra-, penta-, and hexanucleotides with color codes red, blue, green, orange, and purple, respectively. The densely placed spots in the outer ring represented tract length variations for each repeat motif with specific color code. The di (red spots) and tri (blue spots) showed maximum variations which can be exploited for designing chromosome-specific hypervariable SSR markers.

To study distribution of SSRs on different chromosomes, we characterized all SSR motifs into three major classes, i.e., classes I (> 30 nt), II (20–30 nt), and III (< 20 nt). For each chromosome, the variation in the three classes of perfect SSRs with regard to the number of repeat units is presented in Table 4. Total of 337,743 SSRs were considered for classification excluding mononucleotide repeats. The highest number of motifs (301,684; 82.59%) belonged to class III, followed by class II (31,056; 8.50%) and class I (5,003; 1.37%) across all eight chromosomes. The overall distribution graph for three major SSR classes for each chromosome revealed, Chm_1, Chm_2, and Chm_3 with higher number of SSRs for three classes, followed by Chm_4, Chm_5, Chm_7, and Chm_6 (Supplementary Figure 3A). Furthermore, the overall distribution of class I SSRs with respect to the number of repeat units for di-, tri-, tetra-, penta-, and hexanucleotides in each chromosomes was examined (Supplementary Figure 3B), and visually confirmed (Figure 1). As evident from these graphs, the total number of SSRs for the three classes (III, II, and I) and their motif types (di- to hexanucleotides) in each chromosome decreased from inside to outside rings of circos graph. We inferred from the intra-chromosomal distribution of these SSRs that dinucleotides (3,488) were more abundant followed by tri- (1,087), tetra- (214), hexa- (136), and pentanucleotides (78), and the proportion was consistent on all chromosomes (Table 4). As observed on all chromosomes, SSR frequency decreased with an increase in the number of repeat units with the exception of hexanucleotides (Supplementary Figure 3B).

**TABLE 4:** Distribution of three major classes of SSRs in different chromosomes of pomegranate *cv.* Tunisia.

**Chromosomes**	**Class I**						**Class II**	**Class III**	**Total**
	**Di**	**Tri**	**Tetra**	**Penta**	**Hexa**	**Total**			
*Chm_1	547	177	34	11	29	798	4,724	50,897	56,419
Chm_2	549	158	31	13	17	768	4,860	46,132	51,760
Chm_3	451	146	21	15	18	651	4,040	39,265	43,956
Chm_4	494	160	26	11	21	712	4,442	42,275	47,429
Chm_5	397	140	31	8	10	586	3,399	33,129	37,114
Chm_6	324	104	26	8	14	476	3,162	29,484	33,122
Chm_7	377	93	21	6	13	510	3,206	30,655	34,371
Chm_8	349	109	24	6	14	502	3,223	29,847	33,572
Total	3,488	1,087	214	78	136	5,003	31,056	301,684	337,743

*Chm-, chromosome.

### Designing of Chromosome-Specific Hypervariable SSR Primers

Based on the genomic distribution of class I SSRs across all chromosomes, we successfully designed primers for 3,839 out of the total of 5,003 SSRs (Supplementary Table 1). In accordance to class I motif content of various chromosomes, the most primers were designed for Chm_1 (628) followed by Chm_2 (591), Chm_4 (564), and so forth. The majority of these primers were specific to dinucleotide motifs (primers 2,825, 73.59%), followed by trinucleotide repeats (698, 18.18%) (Table 5). For experimental validation, we zeroed in on a set of 906 highly variable SSRs targeting ≥ 40 nt tract length from each chromosome through ePCR on four pomegranate genomes (Supplementary Table 2). The majority of SSR primers used for validation were dinucleotides (616) or trinucleotides (228).

**TABLE 5:** Description of chromosome-specific class I SSR markers designed for eight chromosomes of Tunisia.

	**Number of class I (>30 nt) primers**						**Highly variable SSRs (≥40 nt) primers**					
	**Di**	**Tri**	**Tetra**	**Penta**	**Hexa**	**Total**	**Di**	**Tri**	**Tetra**	**Penta**	**Hexa**	**Total**
*Chm_1	459	112	27	9	21	628	108	35	5	1	10	159
Chm_2	444	106	23	5	13	591	92	43	2	2	3	142
Chm_3	353	95	16	11	9	484	86	25	4	4	3	122
Chm_4	415	106	22	9	12	564	87	39	1	1	3	131
Chm_5	330	88	22	8	9	457	77	26	1	1	2	107
Chm_6	255	63	17	5	11	351	54	17	1	0	5	77
Chm_7	305	62	18	6	7	398	58	21	3	1	2	85
Chm_8	264	66	18	6	12	366	54	22	2	1	4	83
Total	2,825	698	163	59	94	3,839	616	228	19	11	32	906

*Chm-, chromosome.

### Distribution of the New SSR Markers on Different Chromosomes

The 906 SSR genomic location was examined (Supplementary Table 2) and placed on chromosomes (Supplementary Figure 6), of which Chm_1 (159 markers), Chm_2 (142), and Chm_4 (131) had higher number of assigned markers, followed by Chm_3 (122), Chm_5 (107), Chm_7 (85), Chm_8 (83), and Chm_6 (77). Interestingly, scatter plots clearly depicted the physical distance (Mb), intervals between SSRs and their tract lengths on each chromosome. Most of the SSR markers remained in the range of 41–50 nt tract length (612), followed by 51–60 nt (172), 61–70 nt (63), and > 71 nt (59) (Table 6). For tract length 41–50 nt, Chm_1 had most markers (111), whereas Chm_8 had the least number of markers (51). Similarly, for tract length 51–60 nt, Chm_1 had most markers (30) and Chm_6 least markers (14). Concerning the SSR tract length 61–70 nt, three chromosomes, Chm_2 (13), Chm_1 (11), and Chm_3 (11), had higher numbers than Chm_8(4). However, with respect to tract length > 71 nt, Chm_5 had most markers (11), followed by Chm_2 (9), Chm_3 (8), or Chm_1 and Chm_4 (7), Chm_7 and Chm_8 (6), and Chm_6 (5). It is also interesting to note that Chm_7 had highest track length markers (189 bp), followed by Chm_5 (168 bp), Chm_8 (126 bp), Chm_3 (124 bp), Chm_4 (118 bp), Chm_2 (112 bp), Chm_6 (110 bp), and Chm_1 (88 bp) (Supplementary Figure 6).

**TABLE 6:** Classification of 906 highly variable chromosome-specific SSR markers based on their tract lengths.

**Tract length (nt)**	*Chm_1	**Chm_2**	**Chm_3**	**Chm_4**	**Chm_5**	**Chm_6**	**Chm_7**	**Chm_8**	**Total primers**
41–50	111	103	81	92	64	53	57	51	612
51–60	30	17	22	26	25	14	16	22	172
61–70	11	13	11	6	7	5	6	4	63
71–189	7	9	8	7	11	5	6	6	59
Total	159	142	122	131	107	77	85	83	906

*Chm-, chromosome.

### Construction of a High-Density SSR-Based Physical Map for Pomegranate

**FIGURE 2:**
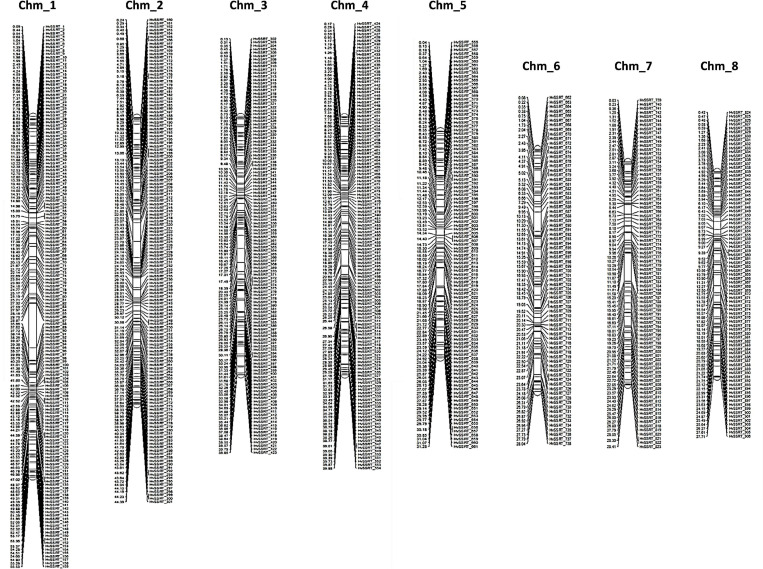
A high-density map of 906 highly variable SSR markers Tunisia (HvSSRT) showing their physical locations on eight chromosomes of pomegranate *cv.* Tunisia.

The physical position and start positions of 906 HvSSRTs on each chromosome, used to generate a high-density physical map (Figure 2) showed that Chm_1 carried the highest number of markers (159), followed by Chm_2 (142), Chm_4 (131), Chm_3 (122), Chm_5 (107), Chm_7 (85), Chm_8 (83), and Chm_6 (77).

### ePCR Validation of the Identified SSRs Across Four Genomes

To assess the amplification efficiency and specificity of the SSRs, initially 3,839 class I SSR primer pairs were tested on ‘Tunisia’ genome through *in silico* analysis. We validated equal proportions of primers across eight chromosomes, and the SSRs produced one to > 3 alleles in ‘Tunisia’ genome (Supplementary Table 3a). A total of 1,165 (30.34%) primers yielded a single amplicon of expected size, whereas 1,263 (32.90%) primers had two alleles, and 1,254 (32.66%) and 157 (4.09%) primers produced three and > 3 alleles, respectively. Furthermore, to validate SSRs with tract lengths of > 40 nt, located on physical map, *in silco* PCR analysis for 906 primers was performed on all the four genome assemblies (‘Dabenzi,’ ‘Taishanhong,’ ‘AG2017,’ and ‘Tunisia’). Notably, we validated all 906 (100%) primers in ‘Tunisia’ genome to that of 853 (94.15%), 832 (91.83%), and 545 (60.15%) in ‘Dabenzi,’ ‘Taishanhong,’ and ‘AG2017’ genomes, respectively (Supplementary Table 3a). A total of 289 (‘Tunisia’), 277 (‘Dabenzi’), 264 (‘Taishanhong’), and 183 (‘AG2017’) SSRs showed single-locus amplification.

To show the informativeness of these chromosome-specific SSRs, we selected a subset of 289 that was validated in all four genomes. The various amplicons detected through ePCR for these 289 primer pairs across the four genomes were recorded to further compute the marker parameters (Supplementary Table 3b). Of these, 265 (91.70%) SSRs were polymorphic across the four genomes. A total of 719 alleles were obtained across the all eight chromosomes. The Na per locus ranged from 2 to 4, with an average of 2.49 alleles/loci (Supplementary Table 4). The MAF per locus varied between 0.50 and 0.88, with an average of 0.74. With an average of 0.46, the PIC values had a range from 0.25 to 0.80. Out of 289 HvSSRT primer pairs validated, 144 SSRs had PIC values ≥ 0.50. For the four genomes tested, the average Shannon information index was 0.66.

We compared all marker parameters at the chromosome level (Table 7). Among eight chromosomes, Chm_8 had lower number of polymorphic markers (25) and higher average value of Ne (1.83), Shannon’s information index (*I* = 0.72), and PIC (0.51). However, Chm_5 also showed lower number of polymorphic markers (26) and lower average value Ne (1.54), *I* (0.51), and PIC (0.35). With respect to PIC value remaining, all six chromosomes (Chm_1, Chm_2, Chm_3, Chm_4, Chm_6, and Chm_7) had mean PIC values of 0.44–0.48.

**TABLE 7:** Chromosome-specific marker statistics for 289 highly variable SSR primer pairs assayed through ePCR across the four pomegranate genotypes based on their genome sequences.

**Chromosome**	**TNP**	**TPP**	**Na**	**MAF**	**Ne**	***I***	***Ho***	***He***	**PIC**
Chm_1	55	48	2.40	0.75	1.71	0.63	0.50	0.38	0.44
Chm_2	42	39	2.52	0.73	1.75	0.68	0.54	0.40	0.47
Chm_3	32	31	2.69	0.72	1.78	0.71	0.56	0.42	0.48
Chm_4	41	40	2.59	0.74	1.75	0.69	0.53	0.41	0.48
Chm_5	35	26	2.17	0.80	1.54	0.51	0.40	0.30	0.35
Chm_6	30	29	2.67	0.73	1.73	0.70	0.53	0.41	0.47
Chm_7	28	27	2.39	0.72	1.73	0.66	0.55	0.41	0.47
Chm_8	26	25	2.54	0.70	1.83	0.72	0.60	0.44	0.51
Total	289	265	2.50	0.74	1.73	0.66	0.53	0.39	0.46

*TNP, total number of primers; *TPP*, total number of polymorphic primers; *Na*, number of alleles; *MAF*, major allele frequency; *Ne*, number of effective alleles; *I*, Shannon’s Information Index; *Ho*, observed heterozygosity; *He*, expected heterozygosity; *PIC*, polymorphic information content.

### PCR Amplification and Polymorphism

**FIGURE 3:**
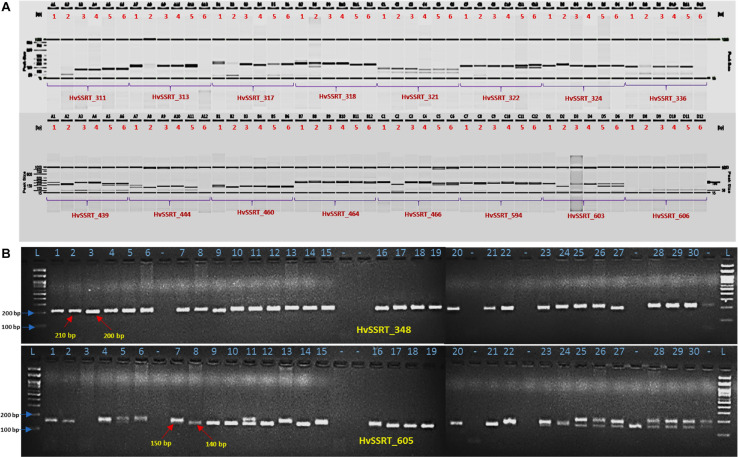
Allelic variations revealed by HvSSRT markers when assayed on six pomegranate genotypes using fragment analyzer **(A)**, HvSSRT markers 348 and 605 on 30 pomegranate genotypes on 3% metaphor gels **(B)** (Lanes 1–6, six sets of pomegranate genotypes, i.e., ‘Bhagawa’, ‘Daru 17,’ ‘Mridula,’ P-23, IC 318728, and IC 318790, L-100 bp DNA ladder for lanes 1–30 pomegranate genotypes as listed in Table 1, genotypes in lane –, were excluded from the analysis).

**FIGURE 4:**
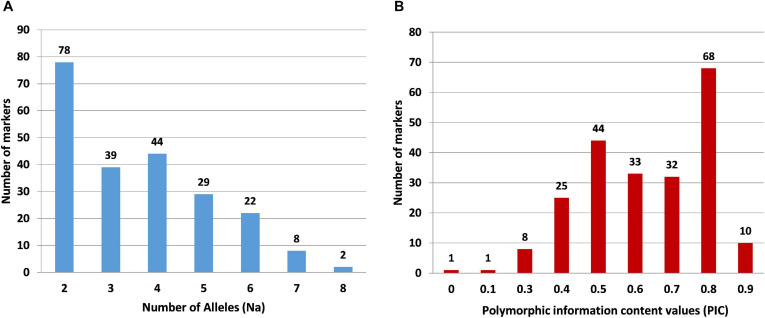
Frequency distribution of SSR markers in six pomegranate genotypes with respect to number of alleles **(A)** and PIC values **(B)**.

For wet-lab validation of the SSRs, we initially screened 235 primer pairs on six pomegranate genotypes. As a result, 225 (94.5%) yielded the amplicons of expected size, whereas 10 remaining primer pairs did not show any amplification. A total of 221 (98.2%) SSRs were polymorphic across six pomegranate genotypes, one marker (HvSSRT_416) remained monomorphic, and three SSRs (HvSSRT_258, HvSSRT_702, and HvSSRT_900) amplified only in one genotype (Supplementary Table 5). A representative gel image illustrating the SSR profiles of pomegranate genotypes is presented in Figures 3A,B. Using SSR markers, we detected 797 alleles among six genotypes as listed in “MATERIALS AND METHODS” (Supplementary Table 5). The Na for loci ranged from 1 to 8, with an average of 3.59. The *Ho* and the *He* for each locus ranged from 0 to 1 (with a mean of 0.58) and 0 to 0.86 (with a mean of 0.61), respectively, and the Shannon’s information index ranged from 0.00 to 2.02 with a mean of 1.08. PIC ranged from 0 (for HvSSRT_416) to 0.96 (HvSSRT_891) with a mean value of 0.68. A total of 187 markers had PIC values ≥ 0.5 (Figure 4B). It is interesting to note that 68 SSRs had PIC values ≥ 0.80. The frequency analysis of HvSSR markers with respect to number of alleles showed that 78 markers had two alleles while eight alleles were obtained for two markers (Figure 4A).

### Assessment of the Genetic Relationships

A subset of 16 HvSSRs located on eight chromosomes, selected randomly from each chromosome based on their clear amplification profiles observed on six genotypes, were tested on 30 pomegranate genotypes (Table 1), and a total of 34 alleles were detected with an average of 2.13 alleles across the pomegranate genotypes. The *Ho* ranged from 0 to 0.52, with an average number of 0.23. The PIC values ranged from 0.33 to 0.60, with an average of 0.48 (Supplementary Table 6). The mean Shannon’s information index at 0.69 revealed moderate diversity among the genotypes.

**FIGURE 5:**
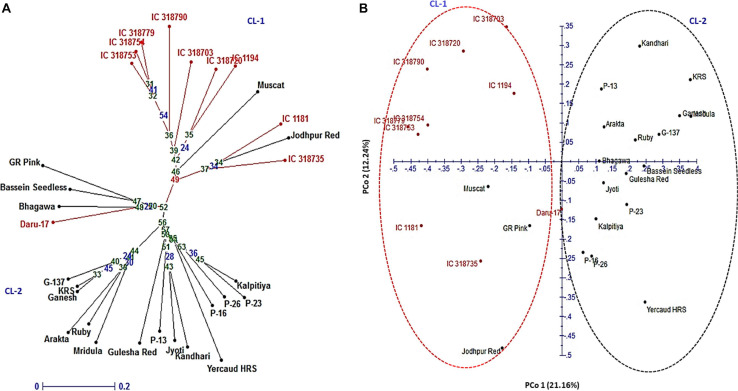
Genetic relationships among 30 pomegranate genotypes based on 16 HvSSRT markers: **(A)** neighbor-joining tree and **(B)** principal coordinate analysis.

In the NJ tree, all 30 pomegranate genotypes were grouped into two major clusters: cluster 1 comprised 11 while cluster 2, 19 genotypes (Figure 5A). Cluster 1 carried 9 wild genotypes with an exceptional placement of one wild genotype (Daru 17) within cluster 2. Cluster 2 had all cultivars (18), with exception of out grouping of two cultivars Muscat and Jodhpur Red in cluster 1. Furthermore, the PCoA assigned 30 genotypes to two major clusters (Figure 5B). The principal coordinates (PCos) 1 and 2 explained 21.16 and 12.24%, respectively, of the total variance among the genotypes and accounted for 33.84% of the total variation. Interestingly, PCo 1 clearly separated two clusters into wild and cultivar groups.

## Discussion

Lack of chromosome-wise details on polymorphic molecular markers in pomegranate has greatly hampered the progress of trait discovery and gene cloning. SSR markers have been proven very useful for genetic analysis, trait mapping, and molecular breeding in several tree species. A large number of genome-wide hypervariable SSRs were recently discovered using draft genome sequence of pomegranate *cv.* Dabenzi (Patil et al., 2020c). However, lack of a chromosome-wide profiling of these hypervariable SSR greatly limits their immediate use in genetic studies.

### The SSR Content of the Tunisia Genome

The present study aimed to offer highly informative chromosome-wise SSR markers in pomegranate. The genomic sequences containing perfect SSRs accounted for 1.74% (5.18 Mb) of total assembled genome, which is slightly higher than observed in the grapevine (0.67%) genome (Portis et al., 2016). The ‘Tunisia’ genome revealed 55,836 (15.28%) SSRs of compound type, which is higher than the ‘Dabenzi’ genome 15,483 (8.92%) (Patil et al., 2020c). Our observation of higher SSR density (1,230.6 SSRs/Mb) in ‘Tunisia’ genome slightly deviated from a previous report that established a negative relationship between genome size and SSR density (Morgante et al., 2002). Such trends were also witnessed through comparing various plant genomes (Portis et al., 2016). By conducting SSR survey in genome sequences of 16 tree species, Xia et al. (2017) found that SSR densities with the tree species may be greater than > 1,200, as in *Theobroma cacao* (1,446) and others. Furthermore, consistent with the observation of Xia et al. (2017), we found hexanucleotide repeats were the most dominant in ‘Tunisia’ genome (55.16%), followed by di-, tetra-, tri-, mono-, and pentanucleotides. The frequency distribution for motif category revealed AT/AG had the highest occurrence, followed by AAAAAT/AAAAAG, A/C, AAT/AAG, AAAT/AAAG, and AAAAT/AAAAG in the ‘Tunisia’ genome. Previously, authors have confirmed the abundance of AT/TA and TTA/TAT/ATT types in the pomegranate genome (Ravishankar et al., 2015). Similarly, Patil et al. (2020c) observed abundance of AT/AT- and AAT/ATT-type motifs in the ‘Dabenzi’ genome. Besides this, we observed abundance of SSR was inversely proportional to the increase in the motif repeat number, and this trend was pronounced for hexa- and tetranucleotide repeat classes, followed by penta-, tri-, di, and mononucleotide repeats, which is in agreement with other reports (Cavagnaro et al., 2010; Shi et al., 2013; Cheng et al., 2016).

### The Intra-Chromosomal Distribution of SSRs

In this study, we found that the number of SSRs and SSR densities vary according to the length of the chromosomes. Consistent with our finding, Portis et al. (2016) also observed greatest number of SSRs (15,212 perfect, 19,282 imperfect) being assigned to the longest LG02 (70.34 Mb) and the lowest (5,181 perfect, 6,518 imperfect) to the shortest LG14 (14.48 Mb) in globe artichoke genome, yielding varying SSR densities of 216.3 to 357.9/Mbp. Similar observations were made in soybean (Ott et al., 2011) and eggplant (Portis et al., 2018). Concerning the intra-chromosomal distribution of motif types in ‘Tunisia,’ hexanucleotide was the most common SSR type followed by dinucleotides, which is in best agreement with earlier report, where hexamers accounted for 55.16% of all motifs, and pentamers were the least abundant type (2.33%) in tree genomes (Xia et al., 2017).

### Chromosome-Specific Distribution of Three Major Classes of SSRs

Examination of distribution of SSR types across chromosomes revealed class III SSRs as the most dominant followed by classes II and I. The relationship between SSR frequency and their repeat number in each chromosomes reported here agreed with previous reports in various plant species, i.e., *Brassica*, *Arabidopsis*, and other angiosperm species (Shi et al., 2013), pepper (Cheng et al., 2016) and globe artichoke (Portis et al., 2016). Furthermore, dinucleotides dominated followed by trinucleotide repeats for class I SSRs in each chromosome. These patterns were also reflected from the genome-wide distribution of the three major classes of SSRs.

### Designing and Distribution of Chromosome-Specific Hypervariable SSR Markers

We designed primers for 3,839 class I SSR markers of which most markers were specific to Chm_1 (628), while Chm_8 (366) and Chm_6 (351) had lower number of markers. Earlier, a high correlation (*R*^2^ = 0.96, *P* < 0.01) was reported between chromosome length and number of SSRs in eggplant chromosomes, with the longest chromosome (E01) containing the highest number of SSRs and the shortest hosting the least (Portis et al., 2018). The present study suggested that for each chromosome, the distribution of markers decreases with increase in tract length, which was also reflected at whole genome level. Singh et al. (2010) also found a decrease in the number of SSR loci with increase in tract length in the rice genome. In pomegranate, dinucleotides dominated for each chromosome (AT) followed by trinucleotide (AAT) motifs as found over whole ‘Tunisia’ genome. Portis et al. (2018) also noticed similar distribution of motif types within individual chromosomes of eggplant which was very similar to the pattern found over the whole eggplant genome with di- and trinucleotide repeats being the most frequent.

### Construction of a High-Density Physical Map Based on HvSSRTs

A high-density physical map with uniform genomic positions and coverage is necessary for conducting high-resolution gene/QTL mapping (Lu et al., 2019). In pomegranate, however, reports on construction of SSR-based linkage map are currently lacking. Here, we built a saturated physical map of pomegranate using 906 HvSSRT markers. Chm_1 showed maximum number of markers, followed by Chm_2, Chm_4, Chm_3, Chm_5, Chm_7, Chm_8, and Chm_6. This reflected a concordance in numbers of markers with length of chromosomes. The SSR distribution pattern suggested nearly even presence of markers on each chromosome, with slight deviation of spare distribution of markers at the middle of chromosomes as compared with distal ends. This high-density physical map could serve as the reference map for analyzing the high-throughput genotyping data for different types of populations and accelerate mapping and breeding applications of different traits in pomegranate. Several reports have shown the application of SSR-based physical map in fine mapping of reported QTLs (Zhao et al., 2017). Genetic and physical maps based on SSR markers in many crops have served as important genomic resources to study collinearity and synteny (Jamshed et al., 2016; Kirungu et al., 2018). Therefore, we assume that the information generated here could definitely provide benefits to the pomegranate researchers for trait mapping.

### ePCR Validation and Identification of Single-Locus SSRs

We performed an *in silico*-simulated PCR to assess SSR polymorphism levels across four pomegranate genome sequences. An initial ePCR with 3,839 class I SSR primer pairs on ‘Tunisia’ chromosomes identified 1,165 (30.34%) produced single allele of expected size. Subsequently, we selected 906 high variable class (> 40 nt) markers for validation and identified 289 primer pairs producing expected product size with single amplicon. The ePCR technique has been applied to validate *in silico*-discovered DNA markers in different plant species including wheat (Han et al., 2015), sesame (Dossa et al., 2017), carrot (Uncu and Uncu, 2020), cucumber (Liu et al., 2015), bitter melon (Cui et al., 2017), and tobacco (Wang et al., 2018).

### Identification of a Set of SSRs With Higher Level of Polymorphism

High level of DNA polymorphism is the most important characteristic of molecular markers. The present study provides a total of 906 (> 40 bp) ePCR-validated SSRs spanning all eight chromosomes of ‘Tunisia.’ Among these, 289 markers showed single ePCR product in the ‘Tunisia’ genome. Of these 289 primer pairs, 277 were validated in ‘Dabenzi,’ 264 in ‘Taishanhong,’ and 183 in ‘AG2017’ through e-mapping with 91.70% being polymorphic and having an average PIC value of 0.46. Further classification of primer pairs based on PIC values led to the identification of 144 SSRs as highly polymorphic (PIC: 0.50 to 0.80), while 121 SSRs (PIC: 0.25–0.46) had moderate level of polymorphism. In our previous study in pomegranate, the 82 HvSSRs identified from draft genome sequence of ‘Dabenzi’ had average PIC value of 0.28, and importantly, 46 HvSSRs (56.10%) had PIC values ≥ 0.4 (Patil et al., 2020c). Taken together, these findings suggest an increase in the SSR polymorphism with an increase in the length of the SSR tracts (Cavagnaro et al., 2010; Bohra et al., 2011).

### Wet-Lab Validation of SSRs and Marker Polymorphism Survey

Wet-lab validation of ePCR-derived 235 HvSSRs revealed 98% (221) polymorphism in six pomegranate genotypes. The PIC observed among six genotypes (ranged from 0 to 0.96 with a mean of 0.68) is higher than the range (0–0.91) reported earlier in 12 pomegranate genotypes (Ravishankar et al., 2015). Earlier, we obtained PIC values between 0.12 and 0.63 for 82 polymorphic SSRs from analysis of eight pomegranate genotypes (Patil et al., 2020c). The differences in SSR allele count and PIC values observed in these studies could possibly be due to use of different genotyping platforms, i.e., agarose, metaphor gels, and automated capillary-based systems (Patil et al., 2020c).

### SSR-Based Diversity Analysis of a Broader Set of Pomegranate Germplasm

The utility of the new SSRs for pomegranate genetic improvement was evident from the diversity estimation of 30 pomegranate genotypes. Analysis with 16 HvSSRs representing eight chromosomes generated a total of 34 alleles with an average PIC value of 0.48. The current results concur with our earlier findings where we obtained 30 alleles having the PIC values between 0.12 and 0.63, following analysis of 46 pomegranate genotypes using 13 HvSSRs derived from ‘Dabenzi’ genome with unknown chromosomal regions (Patil et al., 2020c). Our present results suggested presence of moderate level of genetic diversity among 30 pomegranate genotypes. This could be probably due to low resolution of agarose or metaphor gel for SSR separation and scoring, or due to lesser number of polymorphic alleles among the cultivars examined here (Patil et al., 2020a). The neighbor-joining tree based on 16 HvSSRs grouped the 30 pomegranate genotypes into two distinct clusters representing wild and cultivars groups. These patterns are in concordance with previous clustering patterns observed in pomegranate (Patil et al., 2020a, c). Likewise, a previous analysis of 88 pomegranate accessions with 44 SSRs distinctly separated wild accessions from cultivated types including commercial varieties, local types and introduced accessions (Singh et al., 2015). Considering the higher genetic diversity levels, except Muscat (Oman), all the introduced exotic pomegranate accessions like Kalpitiya (Sri Lanka), GR Pink and Gulesha Red (Russia), and Kandhari (Afghanistan) were distributed randomly in the cluster 2 as compared with the cluster 1 that contained only wild accessions. The resulting clustering patterns agreed with geographical distributions and pedigree relationships. For instance, cluster 1 contained Daru type wild accessions belonging to hill regions of North India including Uttarakhand and Himachal Pradesh.

The close proximity of the genotypes G-137, Ganesh, and Arakta was according to their pedigree. Similarly, the genotypes namely P-23, P-26, P-16, and P-13 representing a selection from Muscat grouped together in a single cluster. In PCoA plot, a total of 33.84% of the variation was explained and the PCo 1 explained 21.16% of variability by clearly separating wild and cultivars groups. In a previous PCoA of 42 diverse pomegranates, axis 1 explained higher proportion of the variance (30.8%) to that of axis 2 (17.95%), and axis 1 had clearly separated the two major clusters (Patil et al., 2020a). Overall, a strong agreement was observed between NJ tree and factorial analysis in our study.

## Conclusion

In the current work, we characterized microsatellites from the survey of entire ‘Tunisia’ genome and validated SSRs that represent eight different chromosomes. A total of 365,279 perfect SSRs were identified. The study provides a large number of chromosome-specific hypervariable (3,839) and highly variable SSRs (> 40 nt, 906) for genotyping applications in pomegranate. We also provide genomic positions of these new SSRs to enable informed selection of most useful SSRs for future use. We first assessed *in silico* amplification of 906 HvSSRs through ePCR and identified 289 SSRs that amplify a single locus. The ePCR approach applied on four pomegranate genome assemblies elucidated 265 SSRs to be polymorphic. We finally confirmed 235 HvSSRs through wet-lab experiment, and 94% of these SSRs were polymorphic. The immediate application of these markers was shown in a panel of 30 pomegranate genotypes. The new set of HvSSRTs developed here could be useful for future gene discovery, genomics-assisted breeding, and germplasm management in pomegranate.

## Data Availability Statement

The original contributions presented in the study are included in the article/Supplementary Material, further inquiries can be directed to the corresponding author/s.

## Author Contributions

PP, NS, and JS designed the research experiments. PP and KR performed the *in silico* analysis to design genome-wide SSR markers. KB and NS contributed in collection of test materials. PP, RM, and DM performed the wet-lab experiments for SSR validation and diversity analysis. PP and AB wrote the original manuscript with assistance of JS. All authors contributed to the article and approved the submitted version.

## Conflict of Interest

The authors declare that the research was conducted in the absence of any commercial or financial relationships that could be construed as a potential conflict of interest.

## Footnotes

1 http://www.ncbi.nlm.nih.gov

## Supplementary Material

Supplementary Figure 1 Frequency distribution of the different SSR motif types in the pomegranate genome *cv.* Tunisia (Supplementary Table 7).

Supplementary Figure 2 Intra-chromosomal distribution of SSRs. Comparison of perfect SSR number/density **(A)** and frequency of mono- to hexanucleotide motifs **(B)** in the eight chromosomes of pomegranate genome *cv.* Tunisia (Supplementary Table 7).

Supplementary Figure 3 Distribution of SSRs across all chromosomes of pomegranate *cv.* Tunisia, frequency of three major classes of SSRs **(A)** and of di- to hexanucleotide motifs **(B)** (Supplementary Table 7).

Supplementary Figure 4 The distribution of SSR repeat lengths for mono- to hexanucleotide motif types across the genome.

Supplementary Figure 5 Chromosome-wise relative abundance of most frequent SSR motif types.

Supplementary Figure 6 Scatter plot depicting chromosome-wise physical distribution of highly variable SSR markers Tunisia (906 HvSSRT) in relation to their track lengths on eight chromosomes of pomegranate *cv.* Tunisia.

Supplementary Table 1 Description of 3,839 class I SSR primer pairs designed based on ‘Tunisia’ genome information.

Supplementary Table 2 Description of 906 highly variable class I SSRs selected for ePCR validation and their physical location and tract lengths across the eight chromosomes of ‘Tunisia’.

Supplementary Table 3 **(a)** Experimental validation of class I SSRs in ‘Tunisia’ genome and highly variable SSRs (906) through ePCR or eMapping across the other three draft genomes of pomegranate cultivars Dabenzi, Taishanhong, and AG2017. **(b)** Allele score for 289 highly variable SSRs validated through ePCR on four different pomegranate genomes.

Supplementary Table 4 Marker statistics computed for 289 HvSSRT markers screened through ePCR on four pomegranate genomes.

Supplementary Table 5 Marker statistics for 222 HvSSRT primer pairs validated through PCR on six pomegranate genotypes.

Supplementary Table 6 Genetic diversity statistics of 16 hypervariable SSR markers Tunisia (HvSSRT) obtained from 30 pomegranate genotypes.

Supplementary Table 7 Input data used for drawing various frequency distribution graphs.
