# Supplementary figures and images for: Comprehensive Characterization and Validation of Chromosome-Specific Highly Polymorphic SSR Markers From Pomegranate (Punica granatum L.) cv. Tunisia Genome
Source: Front Plant Sci. 2021 Mar 16;12:645055. doi: 10.3389/fpls.2021.645055 (PMC8007985; doi:10.3389/fpls.2021.645055)

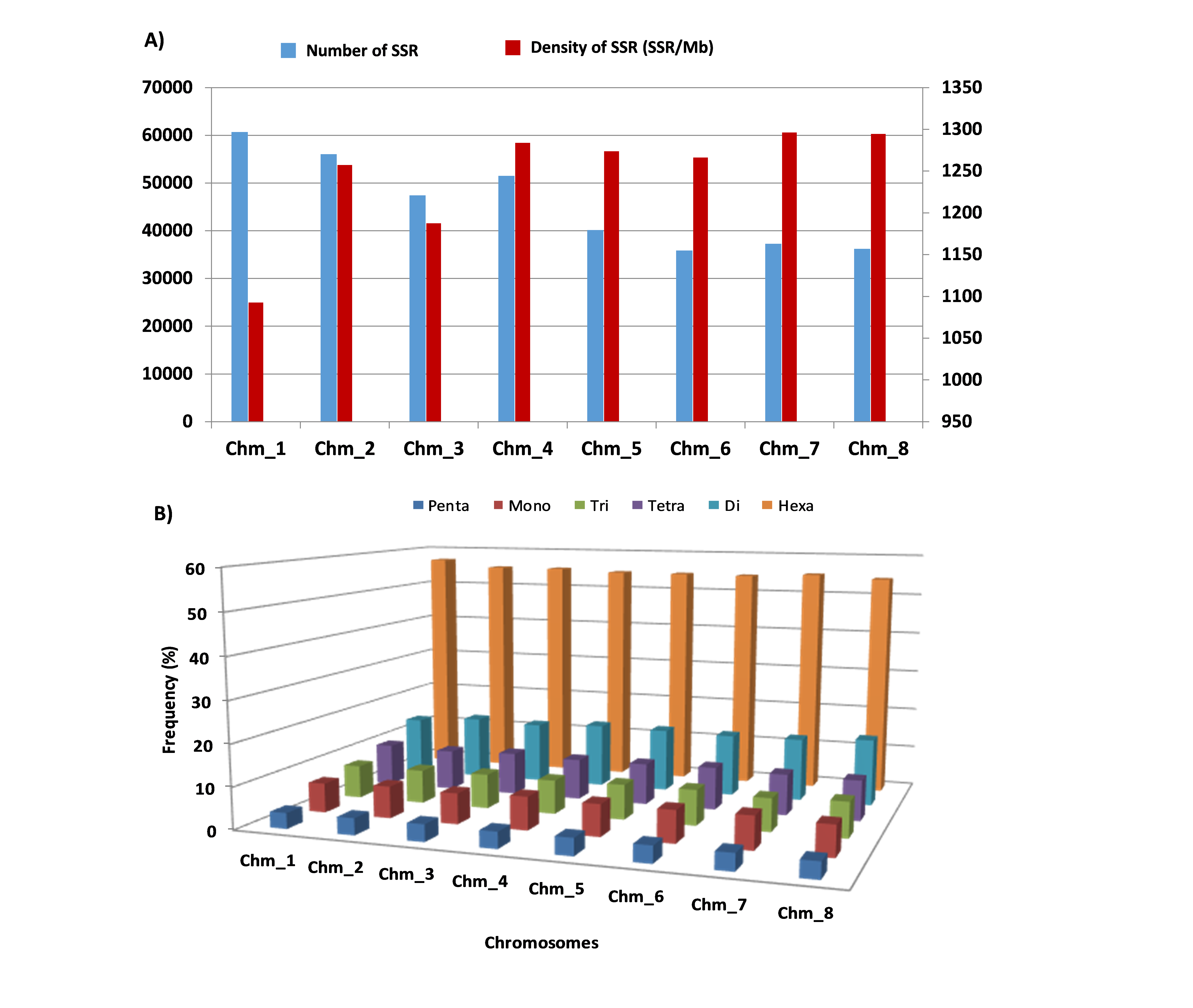

Supplement: Supplementary Figure 1 — Frequency distribution of the different SSR motif types in the pomegranate genome cv. Tunisia (Supplementary Table 7). [file Image_1.TIF]

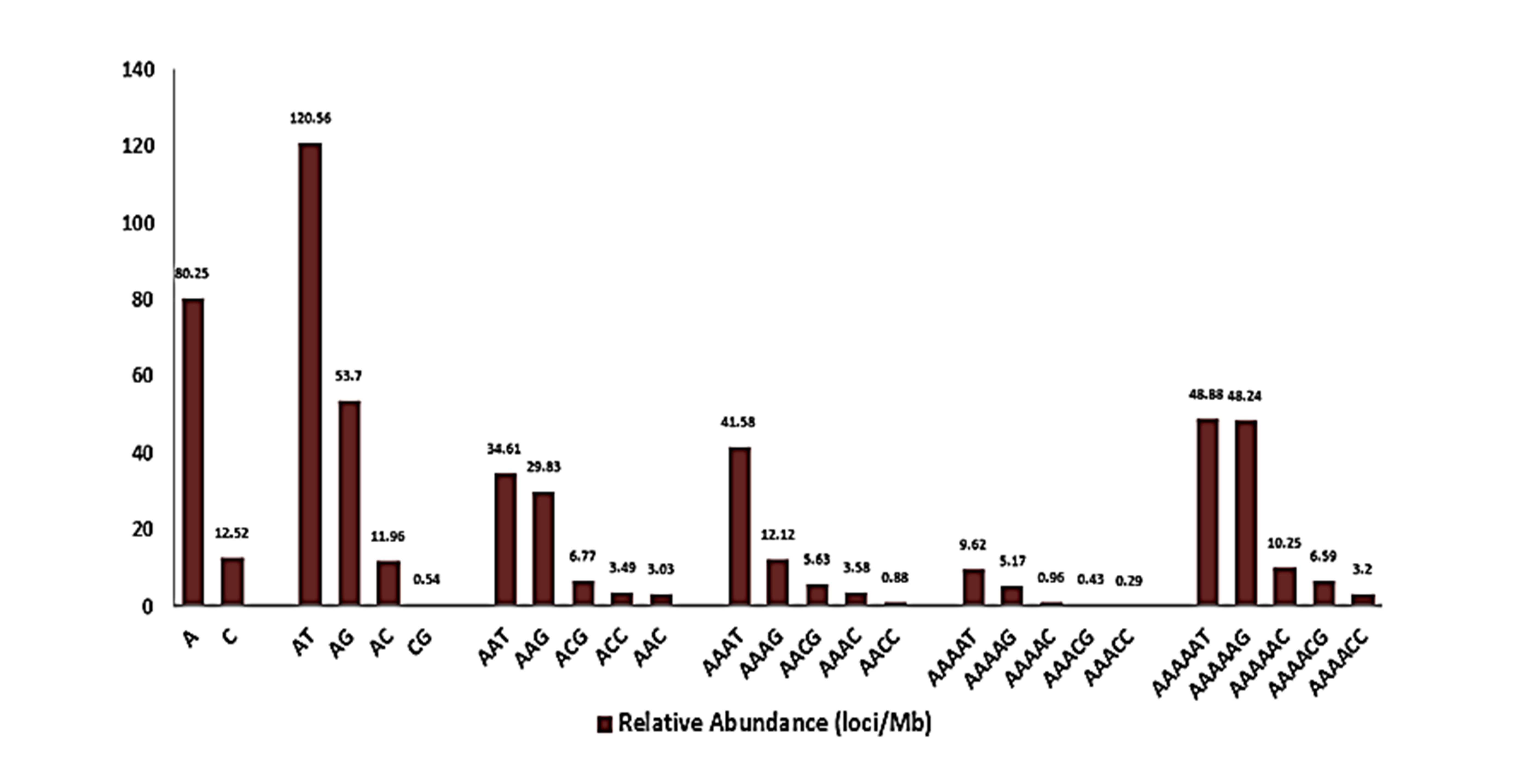

Supplement: Supplementary Figure 2 — Intra-chromosomal distribution of SSRs. Comparison of perfect SSR number/density (A) and frequency of mono- to hexanucleotide motifs (B) in the eight chromosomes of pomegranate genome cv. Tunisia (Supplementary Table 7). [file Image_2.TIF]

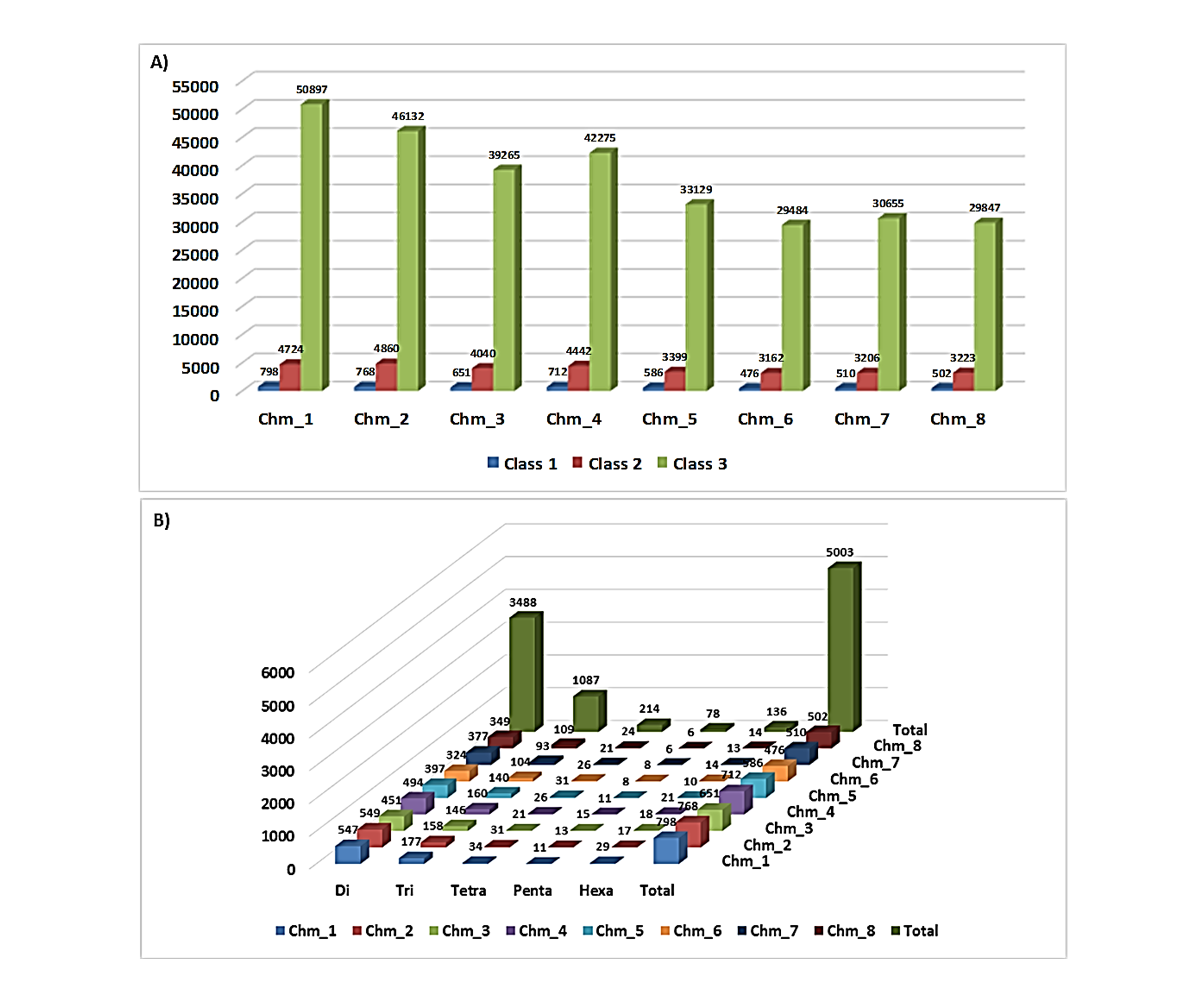

Supplement: Supplementary Figure 3 — Distribution of SSRs across all chromosomes of pomegranate cv. Tunisia, frequency of three major classes of SSRs (A) and of di- to hexanucleotide motifs (B) (Supplementary Table 7). [file Image_3.TIF]

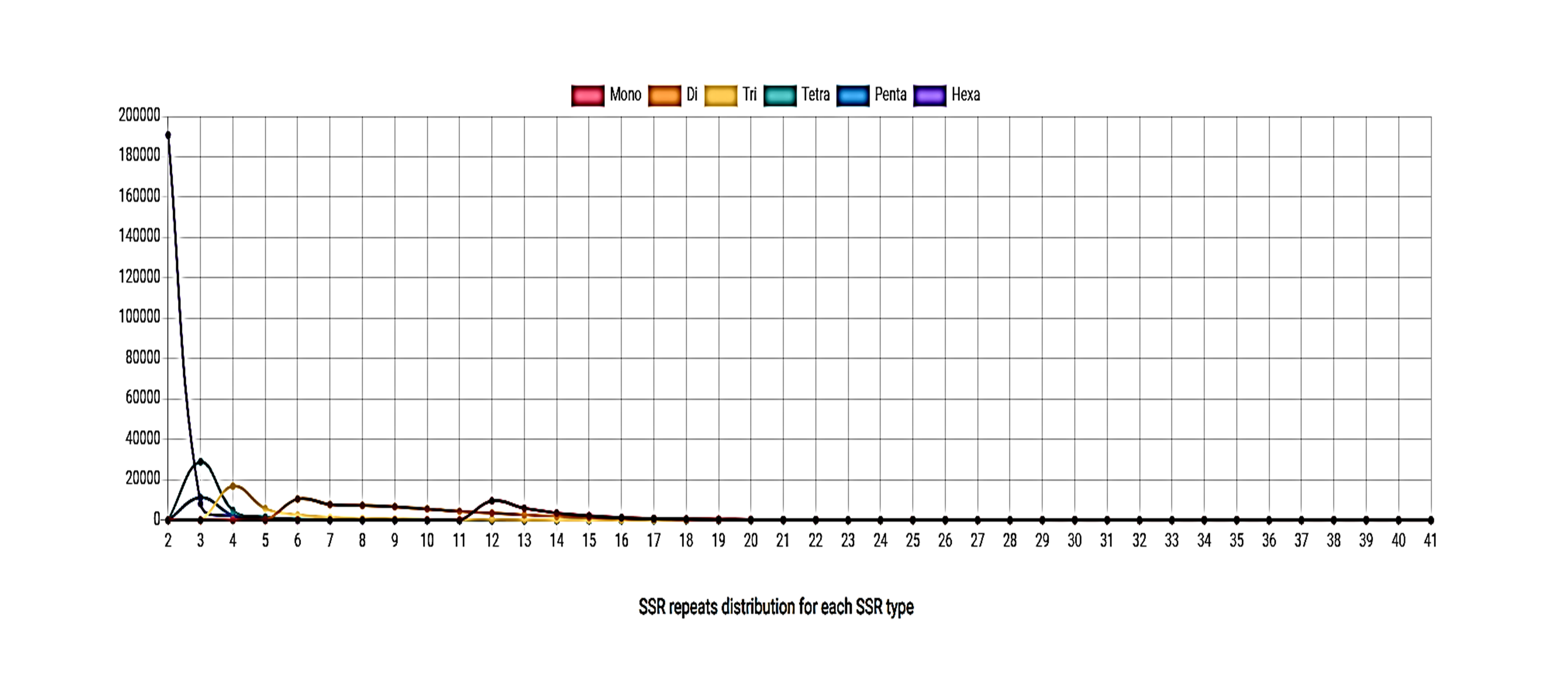

Supplement: Supplementary Figure 4 — The distribution of SSR repeat lengths for mono- to hexanucleotide motif types across the genome. [file Image_4.TIF]

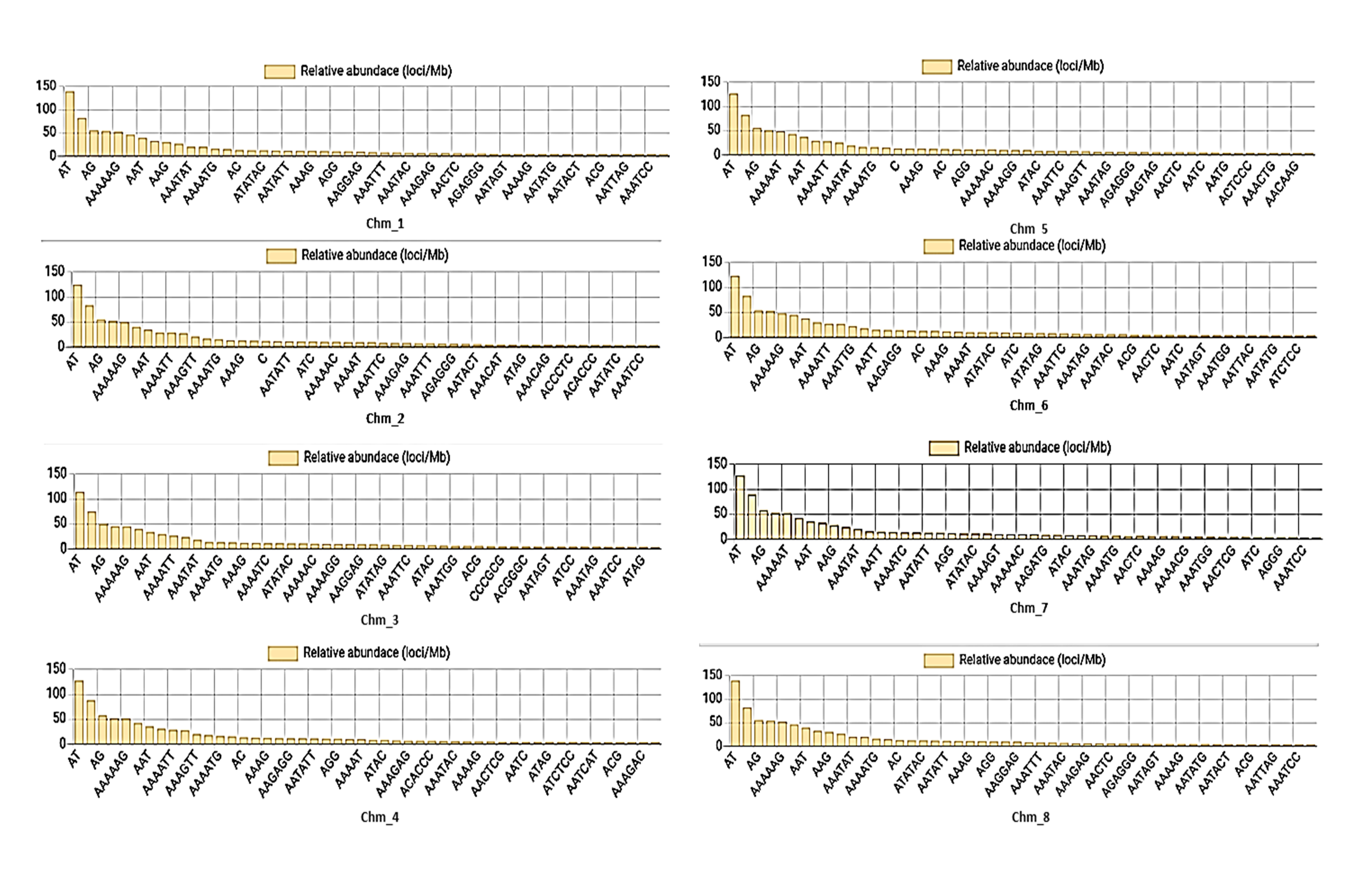

Supplement: Supplementary Figure 5 — Chromosome-wise relative abundance of most frequent SSR motif types. [file Image_5.TIF]

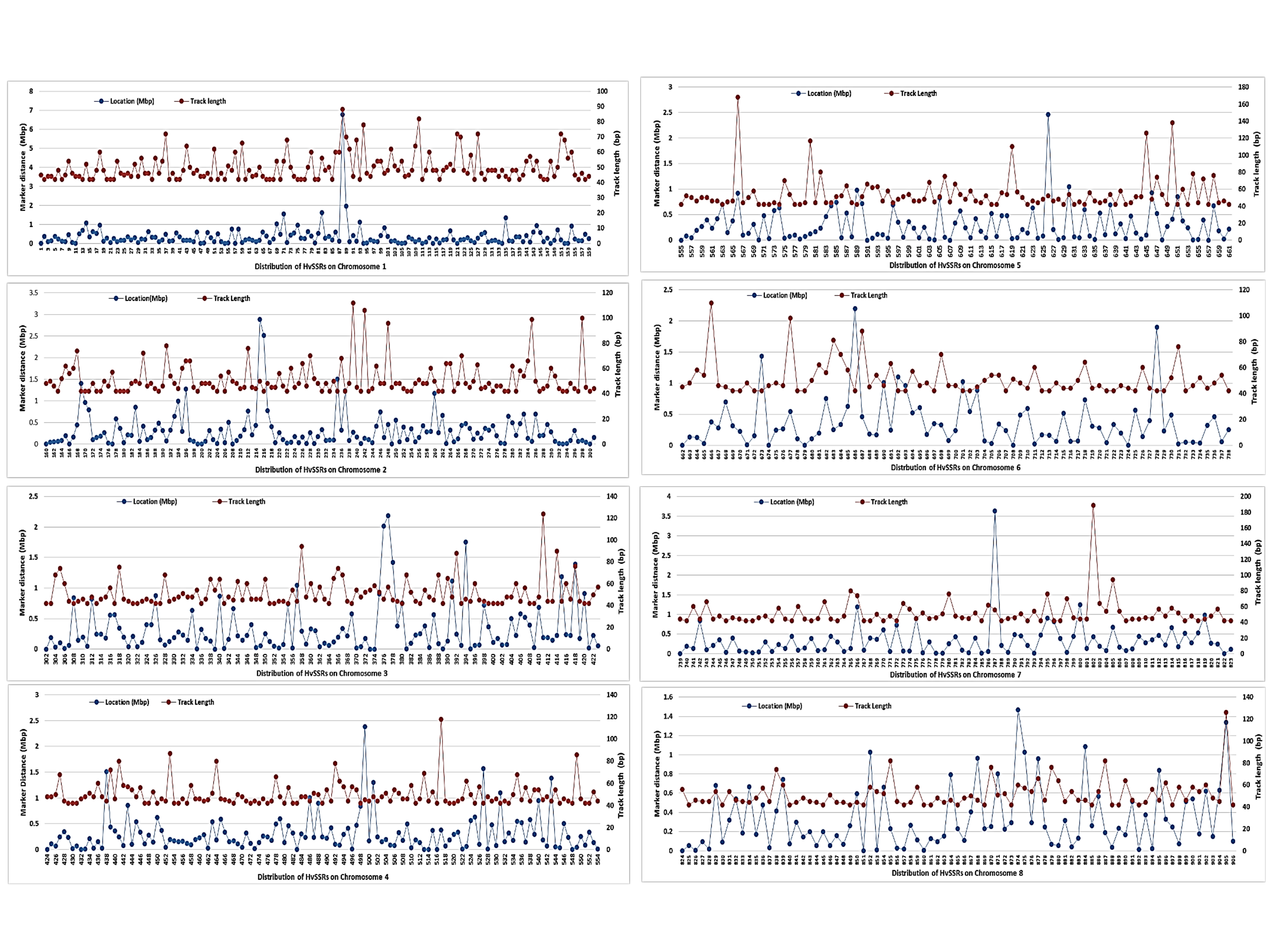

Supplement: Supplementary Figure 6 — Scatter plot depicting chromosome-wise physical distribution of highly variable SSR markers Tunisia (906 HvSSRT) in relation to their track lengths on eight chromosomes of pomegranate cv. Tunisia. [file Image_6.TIF]
