# Supplementary material for: Comprehensive Characterization and Validation of Chromosome-Specific Highly Polymorphic SSR Markers From Pomegranate (Punica granatum L.) cv. Tunisia Genome
Source: Front Plant Sci. 2021 Mar 16;12:645055. doi: 10.3389/fpls.2021.645055 (PMC8007985; doi:10.3389/fpls.2021.645055)
Supplement: Supplementary Table 3 — (a) Experimental validation of class I SSRs in ‘Tunisia’ genome and highly variable SSRs (906) through ePCR or eMapping across the other three draft genomes of pomegranate cultivars Dabenzi, Taishanhong, and AG2017. (b) Allele score for 289 highly variable SSRs validated through ePCR on four different pomegranate genomes. [file Table_3.DOC]

**Supplementary Table S3a.** Experimental validation of class I SSRs in ‘Tunisia’ genome and highly variable SSRs (906) through ePCR or eMapping across the other three draft genomes of pomegranate cultivars Dabenzi, Taishanhong and AG2017

|  |  | **e PCR Validation of Class I SSRs (>30nt) on Tunisia Genome** | | | | |
| --- | --- | --- | --- | --- | --- | --- |
|  |  | **Allele No** | | | | |
|  | **Number of Class I primers designed** | **one** | **two** | **three** | **>three** | **Total (%)** |
| **Chm_1** | **628** | 194 (30.9%) | 202 (32.16%) | 208 (33.12%) | 24 (3.82%) | **100** |
| **Chm_2** | **591** | 164 (27.75%) | 200 (33.84%) | 191 (32.32%) | 36 (6.09%) | **100** |
| **Chm_3** | **484** | 131 (27.07%) | 166 (34.30%) | 155 (32.02%) | 32 (6.61%) | **100** |
| **Chm_4** | **564** | 181 (32.09%) | 172 (30.50%) | 195 (34.57%) | 16 (2.84%) | **100** |
| **Chm_5** | **457** | 143 (31.29%) | 150 (32.82%) | 151 (33.04%) | 13 (2.84%) | **100** |
| **Chm_6** | **351** | 111 (31.62%) | 120 (34.19%) | 105 (29.91%) | 15 (4.27%) | **100** |
| **Chm_7** | **398** | 126 (31.66%) | 125 (31.41%) | 137 (34.42%) | 10 (2.51%) | **100** |
| **Chm_8** | **366** | 115 (31.42%) | 128 (34.97%) | 112 (30.60%) | 11 (3.00%) | **100** |
| **Total** | **3839** | **1165 (30.34%)** | **1263 (32.90%)** | **1254 (32.66%)** | **157 (4.09%)** |  |

|  | **ePCR Validation of Class I**  **highlyvariable SSRs (>40 nt) for genotyping applications** | | | | | | | | | | | | | | | | | | | |
| --- | --- | --- | --- | --- | --- | --- | --- | --- | --- | --- | --- | --- | --- | --- | --- | --- | --- | --- | --- | --- |
|  | **Tunisia Genome** | | | | | **Dabenzi Genome** | | | | | **Taishanhong Genome** | | | | | **AG2017 Genome** | | | | |
|  | **Allele No** | | | | | **Allele No** | | | | | **Allele No** | | | | | **Allele No** | | | | |
|  | **one** | **two** | **three** | **>three** | **Total** | **one** | **two** | **three** | **>three** | **Total** | **one** | **two** | **three** | **>three** | **Total** | **one** | **two** | **three** | **>three** | **Total** |
| **Chm_1** | 55 | 40 | 58 | 6 | **159** | 50 | 44 | 55 | 6 | **155** | 50 | 44 | 56 | 1 | **151** | 39 | 32 | 28 | 2 | **101** |
| **Chm_2** | 42 | 46 | 45 | 9 | **142** | 37 | 49 | 39 | 9 | **134** | 40 | 41 | 46 | 2 | **129** | 23 | 34 | 26 | 1 | **84** |
| **Chm_3** | 32 | 36 | 40 | 14 | **122** | 39 | 35 | 31 | 11 | **116** | 30 | 43 | 35 | 6 | **114** | 22 | 23 | 29 | 2 | **76** |
| **Chm_4** | 41 | 37 | 47 | 6 | **131** | 49 | 35 | 31 | 5 | **120** | 41 | 38 | 38 | 4 | **121** | 26 | 21 | 21 | 2 | **70** |
| **Chm_5** | 35 | 29 | 37 | 6 | **107** | 33 | 32 | 28 | 7 | **100** | 36 | 29 | 25 | 8 | **98** | 23 | 21 | 17 | 4 | **65** |
| **Chm_6** | 30 | 21 | 18 | 8 | **77** | 24 | 23 | 20 | 6 | **73** | 25 | 22 | 19 | 4 | **70** | 12 | 13 | 11 | 7 | **43** |
| **Chm_7** | 28 | 23 | 30 | 4 | **85** | 25 | 24 | 25 | 4 | **78** | 23 | 23 | 25 | 3 | **74** | 21 | 13 | 14 | 3 | **51** |
| **Chm_8** | 26 | 27 | 28 | 2 | **83** | 20 | 30 | 23 | 4 | **77** | 19 | 31 | 24 | 1 | **75** | 17 | 19 | 17 | 2 | **55** |
| **Total** | **289** | **259** | **303** | **55** | **906** | **277** | **272** | **252** | **52** | **853** | **264** | **271** | **268** | **29** | **832** | **183** | **176** | **163** | **23** | **545** |

Note* Chm- chromosome

**b.** Allele score for 289 highly variable SSRs validated through ePCR on four different pomegranate genomes

|  |  | **Primers code/chromosome** | **Tunisia Genome** | **Dabenzi Genome** | **Taishanhong Genome** | **AG2017 Genome** |  |  |
| --- | --- | --- | --- | --- | --- | --- | --- | --- |
| **SSR No** | **Sl.No** | **Chrm_1** | **Allele Size** | **Allele Size** | **Allele Size** | **Allele Size** | **Allele Nos** | **Polymorphism** |
| 1 | 1 | HvSSRT_2 | 200/200 | 204/200 | 203/200 | ?/? | 3 | P |
| 2 | 2 | HvSSRT_6 | 202/202 | 206/202 | 192/202 | 326/202 | 4 | P |
| 3 | 3 | HvSSRT_9 | 225/225 | 225/224 | 225/224 | 225/224 | 2 | P |
| 4 | 4 | HvSSRT_11 | 151/151 | 152/151 | 151/151 | 152/151 | 2 | P |
| 5 | 5 | HvSSRT_13 | 139/139 | 139/139 | 139/139 | 139/139 | 1 | M |
| 6 | 6 | HvSSRT_15 | 171/171 | 172/171 | 172/171 | 171/171 | 2 | P |
| 7 | 7 | HvSSRT_18 | 118/118 | 119/118 | 118/94 | 118/118 | 3 | P |
| 8 | 8 | HvSSRT_20 | 126/126 | 126/126 | 126/126 | ?/? | 1 | M |
| 9 | 9 | HvSSRT_22 | 203/203 | 205/203 | 205/203 | 203/203 | 2 | P |
| 10 | 10 | HvSSRT_24 | 204/204 | 204/199 | 204/204 | 204/204 | 2 | P |
| 11 | 11 | HvSSRT_26 | 181/181 | 181/163 | 181/171 | ?/? | 3 | P |
| 12 | 12 | HvSSRT_39 | 184/184 | 184/179 | 180/184 | 184/184 | 3 | P |
| 13 | 13 | HvSSRT_43 | 212/212 | 212/212 | 216/212 | 212/212 | 2 | P |
| 14 | 14 | HvSSRT_45 | 100/100 | 100/75 | ?/? | 100/75 | 2 | P |
| 15 | 15 | HvSSRT_52 | 184/184 | 185/184 | 185/184 | 184/161 | 3 | P |
| 16 | 16 | HvSSRT_53 | 155/155 | 155/151 | 155/153 | ?/? | 3 | P |
| 17 | 17 | HvSSRT_56 | 215/215 | 213/215 | 212/215 | 215/215 | 3 | P |
| 18 | 18 | HvSSRT_59 | 178/178 | 178/152 | 178/152 | 178/178 | 2 | P |
| 19 | 19 | HvSSRT_63 | 131/131 | 137/131 | 137/131 | 137/131 | 2 | P |
| 20 | 20 | HvSSRT_64 | 214/214 | 170/214 | 170/214 | 214/214 | 2 | P |
| 21 | 21 | HvSSRT_65 | 181/181 | 181/179 | 181/179 | ?/? | 2 | P |
| 22 | 22 | HvSSRT_66 | 220/220 | 220/220 | 220/220 | 220/220 | 1 | M |
| 23 | 23 | HvSSRT_68 | 187/187 | 189/187 | 189/187 | 190/187 | 3 | P |
| 24 | 24 | HvSSRT_70 | 145/145 | 145/145 | 145/145 | 145/145 | 1 | M |
| 25 | 25 | HvSSRT_72 | 174/174 | ?/? | ?/? | ?/? | 1 | M |
| 26 | 26 | HvSSRT_74 | 127/127 | 127/125 | 127/123 | ?/? | 3 | P |
| 27 | 27 | HvSSRT_80 | 153/153 | 156/153 | 156/153 | 156/153 | 2 | P |
| 28 | 28 | HvSSRT_81 | 234/234 | 234/225 | ?/? | ?/? | 2 | P |
| 29 | 29 | HvSSRT_85 | 193/193 | 196/193 | 196/193 | 196/193 | 2 | P |
| 30 | 30 | HvSSRT_87 | 191/191 | 191/191 | 191/191 | ?/? | 1 | M |
| 31 | 31 | HvSSRT_88 | 236/236 | 236/198 | 236/216 | ?/? | 3 | P |
| 32 | 32 | HvSSRT_91 | 182/182 | 182/170 | 182/168 | 182/182 | 3 | P |
| 33 | 33 | HvSSRT_92 | 150/150 | 150/119 | 149/150 | ?/? | 3 | P |
| 34 | 34 | HvSSRT_94 | 220/220 | 220/199 | 220/198 | 220/199 | 3 | P |
| 35 | 35 | HvSSRT_97 | 108/108 | 123/108 | 111/108 | 117/108 | 4 | P |
| 36 | 36 | HvSSRT_100 | 139/139 | 139/130 | 139/134 | 139/135 | 4 | P |
| 37 | 37 | HvSSRT_101 | 113/113 | 113/99 | 113/108 | ?/? | 3 | P |
| 38 | 38 | HvSSRT_102 | 201/201 | 201/197 | 201/195 | 201/201 | 3 | P |
| 39 | 39 | HvSSRT_104 | 102/102 | 102/92 | 102/94 | ?/? | 3 | P |
| 40 | 40 | HvSSRT_108 | 100/100 | 100/106 | 100/100 | ?/? | 2 | P |
| 41 | 41 | HvSSRT_110 | 256/256 | 256/242 | 256/255 | 256/256 | 3 | P |
| 42 | 42 | HvSSRT_111 | 169/169 | 171/169 | 169/167 | 169/169 | 3 | P |
| 43 | 43 | HvSSRT_117 | 193/193 | 193/187 | 193/187 | 193/193 | 2 | P |
| 44 | 44 | HvSSRT_118 | 172/172 | 172/172 | 172/170 | ?/? | 2 | P |
| 45 | 45 | HvSSRT_122 | 124/124 | 124/124 | 124/123 | 124/124 | 2 | P |
| 46 | 46 | HvSSRT_123 | 237/237 | 237/229 | 237/230 | 237/230 | 2 | P |
| 47 | 47 | HvSSRT_129 | 149/149 | 137/149 | 149/149 | ?/? | 2 | P |
| 48 | 48 | HvSSRT_137 | 236/236 | 236/232 | 236/232 | 236/236 | 2 | P |
| 49 | 49 | HvSSRT_140 | 208/208 | 208/208 | 208/208 | 208/208 | 1 | M |
| 50 | 50 | HvSSRT_143 | 180/180 | 180/178 | 180/180 | ?/? | 2 | P |
| 51 | 51 | HvSSRT_145 | 146/146 | 146/138 | 146/138 | ?/? | 2 | P |
| 52 | 52 | HvSSRT_147 | 123/123 | 124/123 | 124/123 | ?/? | 2 | P |
| 53 | 53 | HvSSRT_154 | 249/249 | 285/249 | 260/249 | 249/249 | 3 | P |
| 54 | 54 | HvSSRT_157 | 149/149 | 149/128 | 140/149 | ?/? | 3 | P |
| 55 | 55 | HvSSRT_159 | 144/144 | 144/124 | 144/124 | 144/124 | 2 | P |
|  |  | Chrm_2 | Allele Size | Allele Size | Allele Size | Allele Size | Alleles | Polymorphism |
| 56 | 56 | HvSSRT_160 | 227/227 | 227/224 | 227/224 | 227/207 | 3 | P |
| 57 | 57 | HvSSRT_172 | 143/143 | 313/143 | 160/143 | ?/? | 3 | P |
| 58 | 58 | HvSSRT_174 | 206/206 | 206/204 | 271/206 | 314/206 | 4 | P |
| 59 | 59 | HvSSRT_176 | 138/138 | 138/138 | 138/138 | 138/138 | 1 | M |
| 60 | 60 | HvSSRT_179 | 152/152 | 155/152 | 155/152 | 152/152 | 2 | P |
| 61 | 61 | HvSSRT_180 | 180/180 | 184/180 | 184/180 | 180/180 | 2 | P |
| 62 | 62 | HvSSRT_186 | 169/169 | ?/? | 168/169 | ?/? | 2 | P |
| 63 | 63 | HvSSRT_187 | 172/172 | 172/168 | 172/159 | ?/? | 3 | P |
| 64 | 64 | HvSSRT_188 | 158/158 | 158/149 | 161/158 | ?/? | 3 | P |
| 65 | 65 | HvSSRT_189 | 219/219 | 230/219 | 242/219 | 219/219 | 3 | P |
| 66 | 66 | HvSSRT_197 | 123/123 | 123/123 | 123/113 | 123/123 | 2 | P |
| 67 | 67 | HvSSRT_200 | 156/156 | 159/156 | 158/156 | 156/156 | 3 | P |
| 68 | 68 | HvSSRT_207 | 180/180 | 180/167 | 180/174 | 180/180 | 3 | P |
| 69 | 69 | HvSSRT_209 | 163/163 | 163/156 | 163/148 | ?/? | 3 | P |
| 70 | 70 | HvSSRT_210 | 145/145 | 156/145 | 145/144 | ?/? | 3 | P |
| 71 | 71 | HvSSRT_213 | 205/205 | 208/205 | ?/? | 338/205 | 3 | P |
| 72 | 72 | HvSSRT_217 | 132/132 | 132/125 | 132/126 | ?/? | 3 | P |
| 73 | 73 | HvSSRT_222 | 214/214 | 219/214 | 225/214 | 219/214 | 3 | P |
| 74 | 74 | HvSSRT_224 | 165/165 | 165/164 | 165/164 | 165/164 | 2 | P |
| 75 | 75 | HvSSRT_225 | 160/160 | 160/159 | 160/159 | ?/? | 2 | P |
| 76 | 76 | HvSSRT_230 | 128/128 | 130/128 | 131/128 | 131/128 | 3 | P |
| 77 | 77 | HvSSRT_237 | 158/158 | 158/143 | 160/158 | ?/? | 3 | P |
| 78 | 78 | HvSSRT_243 | 103/103 | 103/103 | ?/? | ?/? | 1 | M |
| 79 | 79 | HvSSRT_246 | 146/146 | 150/146 | 150/146 | ?/? | 2 | P |
| 80 | 80 | HvSSRT_248 | 213/213 | ?/? | 193/213 | ?/? | 2 | P |
| 81 | 81 | HvSSRT_249 | 167/167 | ?/? | ?/? | ?/? | 1 | M |
| 82 | 82 | HvSSRT_251 | 178/178 | 178/176 | 184/178 | ?/? | 3 | P |
| 83 | 83 | HvSSRT_254 | 197/197 | 197/197 | 197/194 | 197/194 | 2 | P |
| 84 | 84 | HvSSRT_258 | 200/200 | 200/193 | 200/193 | 200/193 | 2 | P |
| 85 | 85 | HvSSRT_259 | 172/172 | 172/157 | 172/172 | 172/157 | 2 | P |
| 86 | 86 | HvSSRT_269 | 159/159 | 159/150 | 159/153 | 159/150 | 3 | P |
| 87 | 87 | HvSSRT_271 | 223/223 | 227/223 | 224/223 | ?/? | 3 | P |
| 88 | 88 | HvSSRT_272 | 105/105 | 105/105 | 107/105 | ?/? | 2 | P |
| 89 | 89 | HvSSRT_275 | 144/144 | 144/144 | 153/144 | 144/144 | 2 | P |
| 90 | 90 | HvSSRT_276 | 132/132 | 131/132 | ?/? | ?/? | 2 | P |
| 91 | 91 | HvSSRT_278 | 195/195 | 195/194 | 195/180 | 195/195 | 3 | P |
| 92 | 92 | HvSSRT_279 | 179/179 | 176/179 | 179/174 | ?/? | 3 | P |
| 93 | 93 | HvSSRT_282 | 207/207 | 207/179 | 207/205 | 207/207 | 3 | P |
| 94 | 94 | HvSSRT_284 | 126/126 | 126/94 | 126/118 | ?/? | 3 | P |
| 95 | 95 | HvSSRT_290 | 226/226 | 226/224 | 226/216 | 226/226 | 3 | P |
| 96 | 96 | HvSSRT_293 | 234/234 | 235/234 | 240/234 | 234/234 | 3 | P |
| 97 | 97 | HvSSRT_298 | 144/144 | 144/144 | 144/100 | 144/144 | 2 | P |
|  |  | Chrm_3 | Allele Size | Allele Size | Allele Size | Allele Size | Alleles | Polymorphism |
| 98 | 98 | HvSSRT_302 | 215/215 | 215/215 | 212/215 | 215/194 | 3 | P |
| 99 | 99 | HvSSRT_303 | 204/204 | 204/192 | 204/118 | 204/118 | 3 | P |
| 100 | 100 | HvSSRT_311 | 133/133 | 133/124 | 135/133 | ?/? | 3 | P |
| 101 | 101 | HvSSRT_313 | 169/169 | 181/169 | 169/156 | 169/169 | 3 | P |
| 102 | 102 | HvSSRT_314 | 117/117 | 117/115 | 117/115 | ?/? | 2 | P |
| 103 | 103 | HvSSRT_317 | 203/203 | 203/193 | 203/202 | 210/203 | 4 | P |
| 104 | 104 | HvSSRT_318 | 201/201 | 201/189 | 201/197 | 201/201 | 3 | P |
| 105 | 105 | HvSSRT_321 | 129/129 | 132/129 | 129/129 | 129/203 | 3 | P |
| 106 | 106 | HvSSRT_322 | 175/175 | 175/166 | 175/175 | 175/175 | 2 | P |
| 107 | 107 | HvSSRT_324 | 191/191 | 191/172 | 191/172 | 191/170 | 4 | P |
| 108 | 108 | HvSSRT_335 | 169/169 | 169/157 | 169/157 | ?/? | 2 | P |
| 109 | 109 | HvSSRT_336 | 177/177 | 178/177 | 177/161 | 177/161 | 3 | P |
| 110 | 110 | HvSSRT_341 | 167/167 | 167/164 | 167/164 | ?/? | 2 | P |
| 111 | 111 | HvSSRT_342 | 159/159 | 159/157 | 159/159 | 159/159 | 2 | P |
| 112 | 112 | HvSSRT_344 | 186/186 | 296/186 | 186/182 | 186/186 | 3 | P |
| 113 | 113 | HvSSRT_348 | 219/219 | 219/205 | 219/217 | 219/202 | 4 | P |
| 114 | 114 | HvSSRT_349 | 166/166 | 166/158 | 166/162 | 207/166 | 4 | P |
| 115 | 115 | HvSSRT_350 | 205/205 | 205/187 | 205/205 | 205/205 | 2 | P |
| 116 | 116 | HvSSRT_359 | 127/127 | 127/123 | 127/125 | ?/? | 3 | P |
| 117 | 117 | HvSSRT_360 | 204/204 | 204/198 | 204/165 | 233/204 | 4 | P |
| 118 | 118 | HvSSRT_369 | 163/163 | 163/145 | 163/145 | 163/145 | 2 | P |
| 119 | 119 | HvSSRT_375 | 151/151 | ?/? | 151/128 | ?/? | 2 | P |
| 120 | 120 | HvSSRT_377 | 191/191 | 191/179 | 191/173 | 191/191 | 3 | P |
| 121 | 121 | HvSSRT_383 | 183/183 | 183/174 | 183/181 | 183/183 | 4 | P |
| 122 | 122 | HvSSRT_389 | 121/121 | 121/115 | 121/121 | 121/121 | 2 | P |
| 123 | 123 | HvSSRT_393 | 162/162 | 162/147 | 162/162 | ?/? | 2 | P |
| 124 | 124 | HvSSRT_397 | 145/145 | 145/142 | 145/139 | 145/145 | 3 | P |
| 125 | 125 | HvSSRT_408 | 166/166 | 166/141 | 166/142 | 166/142 | 3 | P |
| 126 | 126 | HvSSRT_409 | 178/178 | 178/178 | 178/178 | ?/? | 1 | M |
| 127 | 127 | HvSSRT_413 | 163/163 | 176/163 | 176/163 | ?/? | 2 | P |
| 128 | 128 | HvSSRT_416 | 151/151 | 151/144 | 151/144 | ?/? | 2 | P |
| 129 | 129 | HvSSRT_420 | 117/117 | 117/113 | 117/114 | ?/? | 3 | P |
|  |  | Chrm_4 | Allele Size | Allele Size | Allele Size | Allele Size | Alleles | Polymorphism |
| 130 | 130 | HvSSRT_431 | 175/175 | 175/175 | 172/175 | 175/175 | 2 | P |
| 131 | 131 | HvSSRT_432 | 147/147 | 151/147 | 148/147 | ?/? | 3 | P |
| 132 | 132 | HvSSRT_435 | 172/172 | 172/154 | 175/172 | ?/? | 3 | P |
| 133 | 133 | HvSSRT_437 | 134/134 | 134/128 | 134/134 | ?/? | 2 | P |
| 134 | 134 | HvSSRT_439 | 216/216 | 168/216 | 168/216 | 216/216 | 2 | P |
| 135 | 135 | HvSSRT_444 | 145/145 | 145/120 | 149/145 | 145/145 | 3 | P |
| 136 | 136 | HvSSRT_451 | 227/227 | 227/227 | 227/226 | 227/207 | 3 | P |
| 137 | 137 | HvSSRT_452 | 170/170 | ?/? | 170/168 | ?/? | 2 | P |
| 138 | 138 | HvSSRT_456 | 194/194 | 194/194 | 194/193 | 194/194 | 2 | P |
| 139 | 139 | HvSSRT_460 | 143/143 | 149/143 | 143/126 | 143/123 | 4 | P |
| 140 | 140 | HvSSRT_463 | 215/215 | 216/215 | 216/215 | 215/215 | 2 | P |
| 141 | 141 | HvSSRT_464 | 229/229 | 229/229 | 229/213 | 229/229 | 2 | P |
| 142 | 142 | HvSSRT_465 | 166/166 | 166/156 | 166/156 | ?/? | 2 | P |
| 143 | 143 | HvSSRT_466 | 197/197 | 239/197 | 215/197 | 197/197 | 3 | P |
| 144 | 144 | HvSSRT_470 | 119/119 | 131/119 | 119/107 | 119/119 | 3 | P |
| 145 | 145 | HvSSRT_475 | 151/151 | 151/141 | 151/148 | ?/? | 3 | P |
| 146 | 146 | HvSSRT_478 | 156/156 | 156/156 | 156/144 | ?/? | 2 | P |
| 147 | 147 | HvSSRT_479 | 151/151 | 151/145 | 151/145 | 151/144 | 3 | P |
| 148 | 148 | HvSSRT_480 | 140/140 | 140/134 | 140/136 | ?/? | 3 | P |
| 149 | 149 | HvSSRT_483 | 115/115 | 115/97 | 115/106 | ?/? | 3 | P |
| 150 | 150 | HvSSRT_487 | 173/173 | ?/? | 181/173 | ?/? | 2 | P |
| 151 | 151 | HvSSRT_490 | 124/124 | 124/105 | 124/102 | 124/81 | 4 | P |
| 152 | 152 | HvSSRT_492 | 216/216 | 216/189 | 216/192 | 216/207 | 4 | P |
| 153 | 153 | HvSSRT_493 | 204/204 | 204/180 | 204/190 | ?/? | 3 | P |
| 154 | 154 | HvSSRT_497 | 172/172 | 172/157 | 172/172 | 172/172 | 2 | P |
| 155 | 155 | HvSSRT_504 | 177/177 | 177/177 | 180/177 | 177/177 | 2 | P |
| 156 | 156 | HvSSRT_505 | 161/161 | 161/158 | 168/161 | ?/? | 3 | P |
| 157 | 157 | HvSSRT_509 | 146/146 | 146/142 | 146/142 | ?/? | 2 | P |
| 158 | 158 | HvSSRT_512 | 113/113 | 113/85 | 113/89 | ?/? | 3 | P |
| 159 | 159 | HvSSRT_513 | 250/250 | 250/202 | 250/194 | 250/250 | 3 | P |
| 160 | 160 | HvSSRT_514 | 132/132 | 132/116 | 132/116 | 132/132 | 2 | P |
| 161 | 161 | HvSSRT_517 | 298/298 | 298/238 | 298/247 | 298/298 | 3 | P |
| 162 | 162 | HvSSRT_520 | 188/188 | 188/182 | 188/184 | ?/? | 3 | P |
| 163 | 163 | HvSSRT_521 | 123/123 | 128/123 | 138/123 | 123/123 | 3 | P |
| 164 | 164 | HvSSRT_523 | 193/193 | 193/175 | 193/174 | ?/? | 3 | P |
| 165 | 165 | HvSSRT_524 | 168/168 | 168/155 | ?/? | ?/? | 2 | P |
| 166 | 166 | HvSSRT_529 | 173/173 | 173/173 | 173/173 | ?/? | 1 | M |
| 167 | 167 | HvSSRT_532 | 128/128 | 128/124 | 128/124 | ?/? | 2 | P |
| 168 | 168 | HvSSRT_533 | 155/155 | 155/146 | 155/146 | 155/155 | 2 | P |
| 169 | 169 | HvSSRT_536 | 152/152 | 152/127 | 152/127 | ?/? | 2 | P |
| 170 | 170 | HvSSRT_538 | 153/153 | 153/153 | 153/137 | ?/? | 2 | P |
|  |  | Chrm_5 | Allele Size | Allele Size | Allele Size | Allele Size | Alleles | Polymorphism |
| 171 | 171 | HvSSRT_555 | 169/169 | 169/167 | 187/169 | 169/169 | 3 | P |
| 172 | 172 | HvSSRT_560 | 154/154 | 154/150 | 154/149 | 154/154 | 3 | P |
| 173 | 173 | HvSSRT_565 | 150/150 | 150/129 | 150/129 | ?/? | 2 | P |
| 174 | 174 | HvSSRT_571 | 185/185 | 188/185 | 185/185 | ?/? | 2 | P |
| 175 | 175 | HvSSRT_573 | 179/179 | 179/158 | 179/159 | 179/179 | 3 | P |
| 176 | 176 | HvSSRT_574 | 126/126 | 126/126 | 126/126 | 126/126 | 1 | M |
| 177 | 177 | HvSSRT_579 | 186/186 | 186/186 | 186/186 | 186/186 | 1 | M |
| 178 | 178 | HvSSRT_581 | 136/136 | 141/136 | 142/136 | ?/? | 3 | P |
| 179 | 179 | HvSSRT_587 | 180/180 | 180/162 | 180/161 | 180/175 | 4 | P |
| 180 | 180 | HvSSRT_589 | 181/181 | 183/181 | 183/181 | 181/181 | 2 | P |
| 181 | 181 | HvSSRT_590 | 148/148 | ?/? | ?/? | ?/? | 1 | M |
| 182 | 182 | HvSSRT_592 | 208/208 | 208/194 | 208/194 | ?/? | 2 | P |
| 183 | 183 | HvSSRT_594 | 204/204 | 204/186 | 204/188 | 204/186 | 3 | P |
| 184 | 184 | HvSSRT_600 | 183/183 | 183/181 | 183/181 | ?/? | 2 | P |
| 185 | 185 | HvSSRT_603 | 194/194 | 222/194 | 194/192 | ?/? | 3 | P |
| 186 | 186 | HvSSRT_605 | 156/156 | 156/174 | 165/156 | 156/156 | 3 | P |
| 187 | 187 | HvSSRT_606 | 221/221 | 221/221 | 221/208 | ?/? | 2 | P |
| 188 | 188 | HvSSRT_607 | 134/134 | 134/134 | 134/134 | ?/? | 1 | M |
| 189 | 189 | HvSSRT_608 | 189/189 | 255/189 | 255/189 | 189/189 | 2 | P |
| 190 | 190 | HvSSRT_609 | 171/171 | 171/154 | 171/171 | 171/171 | 2 | P |
| 191 | 191 | HvSSRT_611 | 190/190 | 190/177 | 190/188 | 190/190 | 3 | P |
| 192 | 192 | HvSSRT_615 | 157/157 | 157/157 | 157/157 | 157/157 | 1 | M |
| 193 | 193 | HvSSRT_628 | 207/207 | 207/205 | 207/197 | 207/207 | 3 | P |
| 194 | 194 | HvSSRT_629 | 144/144 | 144/144 | 144/144 | ?/? | 1 | M |
| 195 | 195 | HvSSRT_630 | 147/147 | 147/147 | 147/147 | 147/147 | 1 | M |
| 196 | 196 | HvSSRT_636 | 204/204 | 204/195 | 204/195 | 204/204 | 2 | P |
| 197 | 197 | HvSSRT_639 | 113/113 | 119/113 | 116/113 | 113/101 | 4 | P |
| 198 | 198 | HvSSRT_645 | 233/233 | 233/233 | 233/185 | 233/233 | 2 | P |
| 199 | 199 | HvSSRT_646 | 137/137 | 137/137 | 137/137 | ?/? | 1 | M |
| 200 | 200 | HvSSRT_648 | 228/228 | 234/228 | 228/192 | 228/228 | 3 | P |
| 201 | 201 | HvSSRT_650 | 265/265 | 265/265 | 340/265 | ?/? | 2 | P |
| 202 | 202 | HvSSRT_651 | 177/177 | 186/177 | 189/177 | 186/177 | 3 | P |
| 203 | 203 | HvSSRT_652 | 148/148 | ?/? | ?/? | 148/148 | 1 | M |
| 204 | 204 | HvSSRT_653 | 124/124 | 124/118 | 124/118 | 124/118 | 2 | P |
| 205 | 205 | HvSSRT_661 | 134/134 | 134/134 | 134/126 | ?/? | 2 | P |
|  |  | Chrm_6 | Allele Size | Allele Size | Allele Size | Allele Size | Alleles | Polymorphism |
| 206 | 206 | HvSSRT_665 | 174/174 | 174/172 | 174/150 | 174/174 | 3 | P |
| 207 | 207 | HvSSRT_667 | 125/125 | 125/121 | 125/123 | 125/121 | 3 | P |
| 208 | 208 | HvSSRT_668 | 184/184 | 184/172 | 188/184 | 184/184 | 3 | P |
| 209 | 209 | HvSSRT_669 | 143/143 | 145/143 | 143/130 | 143/143 | 3 | P |
| 210 | 210 | HvSSRT_672 | 163/163 | 163/163 | 163/154 | ?/? | 2 | P |
| 211 | 211 | HvSSRT_680 | 172/172 | 172/172 | 178/172 | ?/? | 2 | P |
| 212 | 212 | HvSSRT_684 | 137/137 | 137/137 | 137/111 | 137/137 | 2 | P |
| 213 | 213 | HvSSRT_686 | 132/132 | 147/132 | 138/132 | ?/? | 3 | P |
| 214 | 214 | HvSSRT_692 | 184/184 | 195/184 | 191/184 | 184/184 | 3 | P |
| 215 | 215 | HvSSRT_695 | 210/210 | 210/194 | 210/194 | ?/? | 2 | P |
| 216 | 216 | HvSSRT_696 | 128/128 | 128/128 | 128/116 | ?/? | 2 | P |
| 217 | 217 | HvSSRT_697 | 115/115 | 115/105 | 116/115 | 115/104 | 4 | P |
| 218 | 218 | HvSSRT_699 | 171/171 | 177/171 | 171/167 | ?/? | 3 | P |
| 219 | 219 | HvSSRT_700 | 192/192 | 192/184 | 195/192 | 192/192 | 3 | P |
| 220 | 220 | HvSSRT_701 | 140/140 | 140/140 | 140/140 | 140/140 | 1 | M |
| 221 | 221 | HvSSRT_702 | 132/132 | 177/132 | 144/132 | 132/132 | 3 | P |
| 222 | 222 | HvSSRT_704 | 127/127 | 127/119 | 127/119 | ?/? | 2 | P |
| 223 | 223 | HvSSRT_711 | 176/176 | 176/162 | 176/162 | ?/? | 2 | P |
| 224 | 224 | HvSSRT_713 | 214/214 | 214/212 | 214/212 | 214/214 | 2 | P |
| 225 | 225 | HvSSRT_714 | 114/114 | 114/108 | 114/108 | 114/107 | 3 | P |
| 226 | 226 | HvSSRT_721 | 162/162 | 162/150 | 162/156 | ?/? | 3 | P |
| 227 | 227 | HvSSRT_724 | 138/138 | 138/138 | 138/136 | ?/? | 2 | P |
| 228 | 228 | HvSSRT_725 | 229/229 | 229/223 | 229/207 | 261/229 | 4 | P |
| 229 | 229 | HvSSRT_727 | 141/141 | 141/139 | 141/139 | ?/? | 2 | P |
| 230 | 230 | HvSSRT_728 | 127/127 | 129/127 | 127/115 | 127/127 | 3 | P |
| 231 | 231 | HvSSRT_729 | 207/207 | 207/200 | 214/207 | 207/207 | 3 | P |
| 232 | 232 | HvSSRT_730 | 216/216 | 226/216 | 216/207 | 216/216 | 3 | P |
| 233 | 233 | HvSSRT_736 | 190/190 | 200/190 | 205/190 | 190/190 | 3 | P |
| 234 | 234 | HvSSRT_737 | 156/156 | 156/117 | 158/156 | ?/? | 3 | P |
| 235 | 235 | HvSSRT_738 | 194/194 | 194/194 | 200/194 | 195/194 | 3 | P |
|  |  | Chrm_7 | Allele Size | Allele Size | Allele Size | Allele Size | Alleles | Polymorphism |
| 236 | 236 | HvSSRT_740 | 126/126 | 127/126 | 127/126 | 126/126 | 2 | P |
| 237 | 237 | HvSSRT_742 | 121/121 | 123/121 | 123/121 | ?/? | 2 | P |
| 238 | 238 | HvSSRT_744 | 154/154 | 154/151 | 154/151 | 154/154 | 2 | P |
| 239 | 239 | HvSSRT_746 | 178/178 | 178/177 | 178/177 | ?/? | 2 | P |
| 240 | 240 | HvSSRT_747 | 238/238 | 238/224 | 238/224 | 238/224 | 2 | P |
| 241 | 241 | HvSSRT_749 | 164/164 | 164/163 | 164/163 | 164/163 | 2 | P |
| 242 | 242 | HvSSRT_750 | 157/157 | 159/157 | ?/? | ?/? | 2 | P |
| 243 | 243 | HvSSRT_751 | 159/159 | 266/159 | 159/124 | ?/? | 3 | P |
| 244 | 244 | HvSSRT_754 | 176/176 | 176/172 | 176/170 | 176/176 | 3 | P |
| 245 | 245 | HvSSRT_759 | 137/137 | 137/135 | 137/134 | 137/137 | 3 | P |
| 246 | 246 | HvSSRT_762 | 158/158 | 158/144 | 158/143 | ?/? | 3 | P |
| 247 | 247 | HvSSRT_773 | 254/254 | 254/244 | 254/244 | ?/? | 2 | P |
| 248 | 248 | HvSSRT_778 | 205/205 | 205/189 | 219/205 | 205/205 | 3 | P |
| 249 | 249 | HvSSRT_782 | 227/227 | 227/222 | 227/227 | 227/227 | 2 | P |
| 250 | 250 | HvSSRT_783 | 132/132 | 132/132 | 132/132 | 132/132 | 1 | M |
| 251 | 251 | HvSSRT_786 | 252/252 | 252/252 | 252/251 | 252/252 | 2 | P |
| 252 | 252 | HvSSRT_787 | 230/230 | 254/230 | 230/211 | 230/230 | 3 | P |
| 253 | 253 | HvSSRT_788 | 221/221 | 221/221 | 223/221 | 221/221 | 2 | P |
| 254 | 254 | HvSSRT_791 | 163/163 | 163/148 | 163/148 | 163/163 | 2 | P |
| 255 | 255 | HvSSRT_799 | 134/134 | 134/133 | 135/134 | ?/? | 3 | P |
| 256 | 256 | HvSSRT_800 | 165/165 | 165/139 | 165/139 | 165/139 | 2 | P |
| 257 | 257 | HvSSRT_804 | 135/135 | 135/129 | 135/127 | ?/? | 3 | P |
| 258 | 258 | HvSSRT_812 | 159/159 | 159/152 | 159/138 | ?/? | 3 | P |
| 259 | 259 | HvSSRT_813 | 108/108 | 108/105 | 108/106 | ?/? | 3 | P |
| 260 | 260 | HvSSRT_818 | 199/199 | 199/195 | 199/195 | 199/199 | 2 | P |
| 261 | 261 | HvSSRT_819 | 231/231 | 231/228 | 231/228 | 231/231 | 2 | P |
| 262 | 262 | HvSSRT_820 | 178/178 | 178/158 | 178/172 | 178/158 | 3 | P |
| 263 | 263 | HvSSRT_823 | 170/170 | 170/150 | 170/156 | 170/150 | 3 | P |
|  |  | Chrm_8 | Allele Size | Allele Size | Allele Size | Allele Size | Alleles | Polymorphism |
| 264 | 264 | HvSSRT_825 | 113/113 | 114/113 | 114/113 | ?/? | 2 | P |
| 265 | 265 | HvSSRT_826 | 144/144 | 144/123 | 144/123 | 144/144 | 2 | P |
| 266 | 266 | HvSSRT_827 | 186/186 | 186/175 | 186/175 | ?/? | 2 | P |
| 267 | 267 | HvSSRT_828 | 179/179 | 179/170 | 179/170 | 179/179 | 2 | P |
| 268 | 268 | HvSSRT_831 | 134/134 | 134/107 | 137/134 | 134/134 | 3 | P |
| 269 | 269 | HvSSRT_834 | 126/126 | 126/124 | 126/122 | ?/? | 3 | P |
| 270 | 270 | HvSSRT_835 | 122/122 | 134/122 | 134/122 | ?/? | 2 | P |
| 271 | 271 | HvSSRT_840 | 183/183 | ?/? | ?/? | 183/182 | 2 | P |
| 272 | 272 | HvSSRT_843 | 236/236 | 236/220 | 236/220 | 236/235 | 3 | P |
| 273 | 273 | HvSSRT_844 | 162/162 | 162/162 | 162/162 | 162/162 | 1 | M |
| 274 | 274 | HvSSRT_853 | 139/139 | 139/130 | 139/130 | 142/139 | 3 | P |
| 275 | 275 | HvSSRT_866 | 144/144 | 144/136 | 144/136 | ?/? | 2 | P |
| 276 | 276 | HvSSRT_867 | 180/180 | 180/168 | 180/170 | 180/170 | 3 | P |
| 277 | 277 | HvSSRT_868 | 180/180 | 180/163 | 180/171 | 180/180 | 3 | P |
| 278 | 278 | HvSSRT_869 | 176/176 | 176/157 | 176/175 | 176/157 | 3 | P |
| 279 | 279 | HvSSRT_871 | 202/202 | 202/195 | 202/195 | ?/? | 2 | P |
| 280 | 280 | HvSSRT_874 | 161/161 | 161/146 | 161/154 | ?/? | 3 | P |
| 281 | 281 | HvSSRT_876 | 138/138 | 207/138 | 138/112 | ?/? | 3 | P |
| 282 | 282 | HvSSRT_879 | 188/188 | 188/188 | 188/184 | 188/188 | 2 | P |
| 283 | 283 | HvSSRT_882 | 143/143 | 143/139 | 145/143 | ?/? | 3 | P |
| 284 | 284 | HvSSRT_889 | 121/121 | 129/121 | ?/? | ?/? | 2 | P |
| 285 | 285 | HvSSRT_891 | 218/218 | 218/214 | 218/214 | 230/218 | 3 | P |
| 286 | 286 | HvSSRT_893 | 201/201 | 205/201 | 201/189 | 201/201 | 3 | P |
| 287 | 287 | HvSSRT_897 | 167/167 | 179/167 | 179/167 | 179/167 | 2 | P |
| 288 | 288 | HvSSRT_898 | 138/138 | 138/132 | 138/134 | ?/? | 3 | P |
| 289 | 289 | HvSSRT_900 | 167/167 | 167/153 | 167/163 | 167/131 | 4 | P |
|  |  |  |  |  |  |  |  |  |
| Note* | P | Polymorphic |  |  |  |  |  |  |
|  | M | Monomorphic |  |  |  |  |  |  |
