# Supplementary material for: Comprehensive Characterization and Validation of Chromosome-Specific Highly Polymorphic SSR Markers From Pomegranate (Punica granatum L.) cv. Tunisia Genome
Source: Front Plant Sci. 2021 Mar 16;12:645055. doi: 10.3389/fpls.2021.645055 (PMC8007985; doi:10.3389/fpls.2021.645055)
Supplement: Supplementary Table 6 — Genetic diversity statistics of 16 hypervariable SSR markers Tunisia (HvSSRT) obtained from 30 pomegranate genotypes. [file Table_6.DOCX]

**Supplementary Table S6.** Genetic diversity statistics of 16 hypervariable SSR markers Tunisia (HvSSRT) obtained from 30 pomegranate genotypes

| **SL.No** | **Primer Name** | **Chromosome**  **location** | **Na** | **Ne** | ***I*** | ***Ho*** | **He** | **PIC** |
| --- | --- | --- | --- | --- | --- | --- | --- | --- |
| **1** | HvSSRT_81 | 1 | 2.00 | 1.52 | 0.52 | 0.00 | 0.34 | 0.35 |
| **2** | HvSSRT_122 | 1 | 2.00 | 1.99 | 0.69 | 0.15 | 0.50 | 0.51 |
| **3** | HvSSRT_222 | 2 | 2.00 | 1.96 | 0.68 | 0.14 | 0.49 | 0.50 |
| **4** | HvSSRT_284 | 2 | 2.00 | 1.69 | 0.60 | 0.36 | 0.41 | 0.42 |
| **5** | HvSSRT_375 | 3 | 2.00 | 1.87 | 0.66 | 0.35 | 0.46 | 0.47 |
| **6** | HvSSRT_348 | 3 | 2.00 | 1.47 | 0.50 | 0.20 | 0.32 | 0.33 |
| **7** | HvSSRT_497 | 4 | 2.00 | 1.99 | 0.69 | 0.29 | 0.50 | 0.51 |
| **8** | HvSSRT_504 | 4 | 2.00 | 1.85 | 0.65 | 0.52 | 0.46 | 0.47 |
| **9** | HvSSRT_592 | 5 | 2.00 | 1.98 | 0.69 | 0.04 | 0.49 | 0.50 |
| **10** | HvSSRT_605 | 5 | 2.00 | 1.90 | 0.67 | 0.43 | 0.47 | 0.48 |
| **11** | HvSSRT_713 | 6 | 2.00 | 1.95 | 0.68 | 0.10 | 0.49 | 0.49 |
| **12** | HvSSRT_721 | 6 | 3.00 | 2.46 | 0.98 | 0.30 | 0.59 | 0.60 |
| **13** | HvSSRT_773 | 7 | 2.00 | 2.00 | 0.69 | 0.29 | 0.50 | 0.51 |
| **14** | HvSSRT_812 | 7 | 2.00 | 1.97 | 0.69 | 0.09 | 0.49 | 0.50 |
| **15** | HvSSRT_826 | 8 | 3.00 | 2.35 | 0.93 | 0.38 | 0.57 | 0.58 |
| **16** | HvSSRT_827 | 8 | 2.00 | 1.98 | 0.69 | 0.04 | 0.49 | 0.50 |
|  |  | **Mean (Total)** | **2.13 (34)** | **1.93** | **0.69** | **0.23** | **0.47** | **0.48** |

**Note*** Na-number of alleles, Ne- number of effective alleles, *I-* Shannon’s Information Index, *Ho*-observed heterozygosity, *He-* expected heterozygosity, PIC- polymorphic information content
