# Supplementary material for: Comprehensive Characterization and Validation of Chromosome-Specific Highly Polymorphic SSR Markers From Pomegranate (Punica granatum L.) cv. Tunisia Genome
Source: Front Plant Sci. 2021 Mar 16;12:645055. doi: 10.3389/fpls.2021.645055 (PMC8007985; doi:10.3389/fpls.2021.645055)
Supplement: Supplementary Table 7 — Input data used for drawing various frequency distribution graphs. [file Table_7.docx]

**Supplementary Table 7.** Input data used for drawing various frequency distribution graphs.

**Input file for Supplementary Figure 1**

| **Motif** | **Relative Abundance (loci/Mb)** | **Frequecy** |
| --- | --- | --- |
| A | 80.25 | 23821 |
| C | 12.52 | 3715 |
| AT | 120.6 | 35786 |
| AG | 53.7 | 15941 |
| AC | 11.96 | 3549 |
| CG | 0.54 | 161 |
| AAT | 34.61 | 10272 |
| AAG | 29.83 | 8853 |
| ACG | 6.77 | 2010 |
| ACC | 3.49 | 1036 |
| AAC | 3.03 | 900 |
| AAAT | 41.58 | 12341 |
| AAAG | 12.12 | 3598 |
| AACG | 5.63 | 1670 |
| AAAC | 3.58 | 1064 |
| AACC | 0.88 | 260 |
| AAAAT | 9.62 | 2856 |
| AAAAG | 5.17 | 1534 |
| AAAAC | 0.96 | 285 |
| AAACG | 0.43 | 129 |
| AAACC | 0.29 | 86 |
| AAAAAT | 48.88 | 14509 |
| AAAAAG | 48.24 | 14320 |
| AAAAAC | 10.25 | 3043 |
| AAAACG | 6.59 | 1955 |
| AAAACC | 3.2 | 950 |

**Input file for Supplementary Figure 2(A)**

| **Chromosomes** | **Number of SSR** | **Density (SSR/Mb)** |
| --- | --- | --- |
| Chm_1 | 60,708 | 1092.77 |
| Chm_2 | 56,038 | 1257.43 |
| Chm_3 | 47,442 | 1187.4 |
| Chm_4 | 51,511 | 1283.75 |
| Chm_5 | 40,167 | 1273.87 |
| Chm_6 | 35,868 | 1266.29 |
| Chm_7 | 37,304 | 1296.21 |
| Chm_8 | 36,241 | 1294.62 |

**Input file for Supplementary Figure 2(B)**

| **Chromosome** | **Penta** | **Mono** | **Tri** | **Tetra** | **Di** | **Hexa** |
| --- | --- | --- | --- | --- | --- | --- |
| Chm_1 | 3.75 | 7.06 | 7.98 | 10.12 | 14.12 | 56.97 |
| Chm_2 | 4.00 | 7.63 | 8.06 | 9.74 | 15.38 | 55.19 |
| Chm_3 | 4.07 | 7.35 | 8.31 | 10.25 | 14.81 | 55.20 |
| Chm_4 | 3.90 | 7.92 | 8.13 | 9.85 | 15.61 | 54.59 |
| Chm_5 | 4.08 | 7.60 | 8.41 | 9.97 | 15.40 | 54.54 |
| Chm_6 | 3.98 | 7.66 | 8.50 | 10.32 | 15.11 | 54.44 |
| Chm_7 | 3.99 | 7.86 | 7.91 | 9.88 | 15.26 | 55.10 |
| Chm_8 | 3.87 | 7.36 | 8.48 | 9.73 | 16.22 | 54.33 |
|  | 3.96 | 7.56 | 8.22 | 9.98 | 15.24 | 55.05 |

**Input file for Supplementary Figure 3(A)**

| **Chromosome** | **Class 1** | **Class 2** | **Class 3** |
| --- | --- | --- | --- |
| Chm_1 | 798 | 4724 | 50897 |
| Chm_2 | 768 | 4842 | 46132 |
| Chm_3 | 651 | 4040 | 39265 |
| Chm_4 | 712 | 4442 | 42275 |
| Chm_5 | 586 | 3399 | 33129 |
| Chm_6 | 476 | 3162 | 29484 |
| Chm_7 | 510 | 3206 | 30655 |
| Chm_8 | 502 | 3223 | 29847 |
| Total | 5003 | 31038 | 301684 |

**Input file for Supplementary Figure 3(B)**

| **Chromosome** | **Di** | **Tri** | **Tetra** | **Penta** | **Hexa** | **Total** |
| --- | --- | --- | --- | --- | --- | --- |
| Chm_1 | 547 | 177 | 34 | 11 | 29 | 798 |
| Chm_2 | 549 | 158 | 31 | 13 | 17 | 768 |
| Chm_3 | 451 | 146 | 21 | 15 | 18 | 651 |
| Chm_4 | 494 | 160 | 26 | 11 | 21 | 712 |
| Chm_5 | 397 | 140 | 31 | 8 | 10 | 586 |
| Chm_6 | 324 | 104 | 26 | 8 | 14 | 476 |
| Chm_7 | 377 | 93 | 21 | 6 | 13 | 510 |
| Chm_8 | 349 | 109 | 24 | 6 | 14 | 502 |
| Total | 3,488 | 1087 | 214 | 78 | 136 | 5,003 |
